# Fertility desires and utilization of an integrated HIV care and family planning services among young women living with HIV in semi-rural Uganda: A cross sectional study

**DOI:** 10.1371/journal.pone.0349072

**Published:** 2026-07-24

**Authors:** Edward Kumakech, Deo Benyumiza, Marvin Musinguzi, Wilfred Inzama, Doryn Ebong, James Okello, Lydia Kabiri, Jasper Watson Ogwal-Okeng

**Affiliations:** 1 Department of Nursing, Faculty of Nursing and Midwifery, Lira University, Lira, Uganda; 2 Department of Midwifery, Faculty of Nursing and Midwifery, Lira University, Lira, Uganda; 3 Department of Community Health, Faculty of Public Health, Lira University, Lira, Uganda; 4 Department of Obstetrics and Gynaecology, Faculty of Medicine, Lira University, Lira, Uganda; 5 Department of Obstetrics and Gynaecology, Lira Regional Referral Hospital, Lira, Uganda; 6 Department of Nursing, School of Health Sciences, College of Health Sciences, Makerere University, Kampala, Uganda; 7 Department of Pharmacology, Faculty of Medicine, Lira University, Lira, Uganda; No institution, UNITED KINGDOM OF GREAT BRITAIN AND NORTHERN IRELAND

## Abstract

**Background:**

Little is known about the fertility desires among young women living with HIV (YWLHIV) in sub-Saharan African (SSA) settings and its association with their utilization of an integrated HIV care and family planning (FP) services. This study assessed the fertility desires and the utilization of an integrated HIV care and FP services among YWLHIV in Northern Uganda, where limited data exist to inform the integration of contraception, safer conception and prevention of mother to child (PMTCT) of HIV services.

**Methods:**

In a cross-sectional study design, we collected data from YWLHIV attending antiretroviral therapy (ART) clinics in northern Uganda between November 2022 and April 2023. Using an interviewer-administered questionnaire, participants were asked about their fertility desires and the potential associated factors which included the socio-demographic, HIV, ART, sexual and reproductive health (SRH) and access to the community-based SRH resources. They were also asked whether they ever received FP services from the ART clinic facilities where they usually obtain their HIV care. Descriptive statistics for fertility desires and level of utilization of the integrated HIV and FP services were performed. More so, Chi-square test, Fisher’s Exact test, bivariate and multivariable Poisson regression analyses for the associations between the predictor variables and the fertility desires were performed. The 5% significance level and 95% confidence intervals were considered for the measures of fertility desires and the associations.

**Results:**

We recruited 423 YWLHIV with a median age of 22 (IQR 20–24) years. The data revealed a high fertility desire (88.9%) among the YWLHIV. Single women exhibited 24% lower fertility desires than their married counterparts (p = 0.013). Women who did not know of the integrated FP services offered from the ART clinic facilities had 18% lower fertility desires than their counterparts who knew (p = 0.045). Conversely, women with a positive history of planned pregnancies showed 17% higher fertility desires than their counterparts with positive history of unintended pregnancies (p = 0.040). Similarly, current modern contraceptive users had 23% lower fertility desires than their counterparts who were non-current users (p = 0.003). The utilization of an integrated HIV care and FP services was significantly lower (30.1%) among the YWLHIV with fertility desires compared to 48.9% among those without fertility desires (X^2^ 5.991, df 1, p = 0.014).

**Conclusions:**

The study found a high fertility desires but lower utilization of an integrated HIV care and FP services among the YWLHIV in a Ugandan setting. Whereas the factors responsible for the low utilization of an integrated HIV and FP services remains question for future research, being married, having knowledge and receiving modern contraceptive methods from the ART clinic facility, experiences of planned pregnancy and non-current use of modern contraceptives were associated with the higher odds of fertility desires among the YWLHIV. These findings highlight the need to address the low utilization of an integrated HIV care and FP services, marital expectations and modern contraceptive access within the HIV care for YWLHIV with fertility desires.

## Study background

The high population of sexually active and fertility-desiring young women aged 15–24 living with HIV (YWLHIV) is a global public health concern. In 2023, globally, an estimated 1.9 million adolescent girls and young women (AGYW) aged 15–24 years were living with HIV, compared to 1.2 million adolescent boys and young men of the same age bracket [1]. Similarly, 77% of the AGYW who acquired HIV in 2023 live in the sub-Saharan Africa (SSA) and this include the 60% in Eastern and Southern Africa [1]. In Uganda, by 2022, an estimated 119,000 AGYW were living with HIV [2].

Fertility desires refer to a woman’s aspirations, intentions and motivation to bear children. Fertility desires represent a fundamental aspect of family building and social identity. For YWLHIV, these natural fertility desires have been heavily stigmatized and systematically discouraged [3]. This was primarily due to the well-documented fears of transmitting the HIV infection to a male partner or to the child, alongside concerns that HIV infection plus pregnancy would worsen the mother’s health [3]. This discouragement is structurally perpetuated by the traditional vertical or disintegrated healthcare model. In this model, HIV care providers focus solely on HIV disease management. They often view pregnancy as a risk to be prevented. Similarly, family planning (FP) services operate separately without an integrated expertise on safer conception nor HIV disease management.

Consequently, YWLHIV attend a vertical HIV care system that fails to address their dual needs. This leaves their reproductive goals unsupported. This systemic gap not only violates their reproductive rights, it also forces many to pursue pregnancy without the medical guidance necessary to ensure their safety and that of their partners and future children.

Integrated delivery of HIV care and FP services, a recently introduced care model provide opportunities for essential preconception or even antenatal care alongside management of HIV condition and also enable health educational and awareness raising opportunities [4]. Integrated delivery of HIV and FP services may offer prevention of mother-to-child transmission of HIV (PMTCT) services and safer conception counseling. Ultimately, these services support the women’s goal of having desired and healthy children when the time is right.

Despite the health benefits of the integrated HIV and FP service delivery model for the YWLHIV, little was known about the fertility aspirations of the YWLHIV who attend this integrated care model compared to those who do not. Little was also known about their utilization of the integrated HIV and FP services, more so the factors influencing their reproductive decisions.

Multiple interconnected factors drive fertility desires among women in SSA settings. Deeply rooted sociocultural expectations position childbearing as a fundamental feminine role [5]. Similarly, woman’s marital status, age, educational attainment and number of children significantly influence the women’s reproductive decisions [5]. Studies specifically found that YWLHIV who are on antiretroviral therapy (ART), are younger than 30, are married or cohabiting, have attained a secondary education or above and are childless have a higher fertility desires than their counterparts [3,5–8]. Partner’s fertility desires and economic insecurity further reinforces fertility tendencies, as families frequently view children as future sources of labor and social security [3,6]. Health system limitations compound these pressures. In here, inadequate access to modern contraceptive methods constrain women’s ability to implement their fertility preferences [2,7].

For the YWLHIV, reproductive decision-making involves additional medical and social complexities. The pathophysiological effects of HIV infection combined with ART’s pharmacological impacts may alter women’s fertility desires and outcomes. Current evidence remains inconclusive. Some studies suggest that HIV status diminishes childbearing intentions while others report negligible effects [8,12–14]. Similarly, previous studies show conflicting associations between women’s knowledge of the PMTCT services and fertility desires or the reproductive behaviors [15,16]. These inconsistencies may reflect contextual factors unique to high-prevalence settings that warrants further research investigations.

Worse still, previous studies have insufficiently explored the associations between fertility desires and other key factors. This include insufficient research on the associations between women’s knowledge and perceptions of the safer conception methods, attendance at ART clinic facilities offering an integrated HIV and FP services (including contraceptive access and counseling) and lifestyle factors (e.g., alcohol use), access to FP resources and their fertility desires. These critical gaps highlight the need for further research on how health behaviors, service integration and the sexual and reproductive health (SRH) knowledge shape fertility aspirations among the YWLHIV.

Uganda presents a critical case for investigating these dynamics because of the high HIV prevalence. Among young women aged 15–25 years in Uganda, the HIV prevalence stands at 2.5% [17]. Also 90% of the people living with HIV (PLHIV) in Uganda know their status, 94% are on ART and 94% are virally suppressed [2]. This compounds Uganda’s persistently high total fertility rate of 4.5 per woman [17].

We therefore set out to elucidate the fertility desires and the associated factors among the YWLHIV. Understanding the fertility intentions of the YWLHIV can inform safer conception and PMTCT planning and service delivery. Furthermore, results may guide policy initiatives to better integrate HIV care with comprehensive FP services. This will ultimately improve the health outcomes for this vulnerable population.

## Methods

### Conceptual framework

In examining the associations between the health behaviors, service integration, SRH knowledge and fertility desires, this study considered several potential confounding and explanatory variables. These were selected based on the theoretical relevance and the literature as per the STROBE (Strengthening the Reporting of Observational Studies in Epidemiology) recommendations for reporting confounding variables [18].

We considered the women’s sociodemographic characteristics such as age, marital status, educational attainment and income level. This was because having a younger age, being married, having a lower educational attainment and lower wealth status have been associated with an increased fertility desires in many previous studies [5,8,19].

We considered the woman’s HIV disease -related characteristics such as the women’s years since HIV diagnosis and being in a sero-discordant relationship. These markers of HIV disease such as years of HIV positivity and lower CD4 count specifically were associated with the lower odds of fertility aspirations among the YWLHIV in previous studies in Uganda and Kenya [13,20]. We also considered the woman’s ART-specific characteristics such as the duration on ART. The women’s duration on ART was associated with fertility desires with conflicting results [19].

We further considered the woman’s reproductive and obstetric health characteristics such as the woman’s knowledge and perceptions of safer conception methods and their use of modern contraceptive methods. These SRH service related factors have been associated with fertility desires among the YWLHIV in many previous studies [16,21,22]. The woman’s past planned pregnancy experience was also associated with the higher odds for fertility aspirations in a previous study [9]. Being childless or having fewer children than the woman’s desired reproductive goal was considered as a potential predictor of fertility desires consistent with the previous studies [5,13].

We also considered the HIV care and SRH service integration – related factors such as the woman’s exposure to the FP information or counseling from the ART clinic facility, knowledge of the modern contraceptive services offered at the ART clinic facility and her current use of the modern contraceptive methods. These factors were hypothesized to influence the women’s fertility desires consistent with the previous studies [7,23–26].

We also considered some lifestyle factors such as alcohol consumption. Although previous specific studies on the association between alcohol consumption and fertility desires among the YWLHIV could not be found, many previous studies suggest that woman’s alcohol consumption was associated with reduced fertility, fetal loss and still birth in the general population [27,28]. Therefore, considering alcohol consumption in this study provided valuable insights into its association with fertility desires among the YWLHIV in SSA setting Uganda.

### Study design

This study employed a cross-sectional design [29]. The cross-sectional design allowed for the collection and analysis of the data from the YWLHIV population in Lira district and Lira city at a single point in time. This further provides a snapshot of the YWLHIV’s fertility aspiration rate and its predictive factors. The design was quick to execute, cost-effective and relevant to the research questions.

### Study area and setting

This study was conducted in Lira District and Lira City, Northern Uganda. Specifically, across six public health facilities selected out of the 42 health facilities in the area. The participating health facilities included one regional referral hospital, one primary healthcare centre (also known as health centre level IV) and four dispensaries (health centre level IIIs). These facilities were located in both urban and rural parts of the area. These health facilities maintained functional ART clinics with complete, up-to-date registries of the YWLHIV (aged 15–24). This was the criterion that the excluded facilities couldn’t meet. Lira is located about 342 km north of Kampala the capital city of Uganda. It has rural, semi-urban and urban HIV populations. This made it suitable for studying SRH issues.

### Study population

The target population comprised 504 YWLHIV aged 15–24 years. These were the YWLHIV who were registered and receiving ART services at the six selected public health facilities by the time of data collection from 20^th^ November 2022. Of these, 423 formed the sample size for the study which was determined using a standard formula shown below. The YWLHIV who were too sick to withstand the study procedures were excluded from the study.

### Sample size determination

Given the lack of prior data on the fertility aspiration rate among this YWLHIV aged 15–24 in northern Uganda, the sample size for this cross-sectional study on the fertility desire rate among the YWLHIV in northern Uganda was determined using the Kish Leslie formula for a single population proportion [30,31]. The assumptions included a conservative 50% prevalence of fertility desire, 1.96 standard normal deviation corresponding with 95% confidence level from the standard normal distribution curve and a 0.05 margin of error. This gave the minimum required sample size of 384 YWLHIV. This initial sample size was further adjusted for a 10% non-response rate resulting into a final target sample size of 423 YWLHIV.

To address concerns regarding analytical power, post-hoc sensitivity analysis to evaluate the minimum detectable effect sizes for the secondary/ comparative analyses at 80% power and α = 0.05, with the achieved sample of 423 participants revealed the following:

- For estimating a single proportion, the 95% confidence intervals around any observed prevalence have a maximum width of approximately ±4.8%. This confirms that the precision objective was met.
- For comparing two independent subgroups (e.g., users of the integrated HIV and FP services vs. non-users of the integrated HIV and FP services regarding fertility desires) of roughly equal size, the sample had 80% power to detect a difference of approximately 12–13 percentage points between the groups.
- For the regression model, the sample size of 423 comfortably satisfies the standard events-per-variable rule of thumb (at least 10–15 events per predictor). This allowed for fitting a stable multivariable model.

Therefore, while the formal a priori power calculations for each hypothesis would have strengthened the study design, the final sample size of 423 provides adequate precision for the primary descriptive aim and sufficient sensitivity to detect clinically meaningful differences in the exploratory analyses.

### Sampling and participant recruitment

The stratified random sampling [31,32] was employed to ensure representation of the patients from across the three levels (strata) of public health facilities with ART clinics. The three strata included the patients attending the ART clinics at regional referral hospital, level four health centres (or primary healthcare centre) and level three health centres (or dispensary). Each ART clinic facility has ART clinic registers with the list of the YWLHIV aged 15–24 years attending the facility.

A total of six public health facilities with ART clinics were selected for the study namely Lira Regional Referral Hospital, Lira University Hospital, Ober Health Centre, Ogur Health Centre, Amuca Health Centre and Barapwo Health Centre. The ART clinic facility selection was based on the facility having a complete ART register of the YWLHIV with details such as the patient’s name, age, residence, next of kin or treatment supporter’s name, personal phone number, next of kin or treatment supporter’s phone number and date of ART initiation. The ART clinics collectively served 1,771 YWLHIV aged 15–24 as of September 2022 [33].

The list of the YWLHIV aged 15–24 years from the ART clinic at the regional referral hospital, health centre IV facility and health centre III facility served as the three strata for the stratified random sampling procedure. After stratification, the number of the participants selected from each stratum was established using the principle of probability proportionate to the size of the stratum. In this, the stratum with the highest number of patients provided the highest number of participants to the sample size and vice versa [34]. This resulted into 182, 126, and 115 participants from the ART clinic at the regional referral hospital, ART clinic at the health centre IV facility and the ART clinics at the health centre III facilities respectively as described in our previous article [35].

Within each stratum, participants were selected by simple random sampling (flipping a two-sided coin) until the required sample size is reached by stratum and overall. Upon selection and with the assistance from the ART clinic healthcare providers, the selected participants were identified from among the patients attending the ART clinics during the study visits and enrolled into the study. Similarly, with the assistance from the community health workers affiliated to the ART clinics, some selected participants who were not present at the ART clinics during the study visits were invited by phone to come to the ART clinics or were physically visited at their respective homes to participate in the study.

Upon identification, the YWLHIV were included in the study if they (1) were aged 15–24 years, (2) were on ART medications, (3) were residents of Lira (city or district), (4) were sexually active in the past 12 months, (4) had no previous medical diagnosis of infertility, (5) had not undergone total hysterectomy and (6) were able and willing to provide informed consent for the study.

The visits to homes by the community health workers enabled the study participation of some of the selected YWLHIV who could not be accessed by phone. It also enabled study participation of the YWLHIV who were not yet due for scheduled revisit to the ART clinic for drug refill at the time of study.

The process of participant sampling from the ART clinic registers by stratum, identification with the help of the ART clinic workers and enrolment into the study were repeated until the required sample size was reached by stratum and overall. To attain the 423 sample size, a total of 523 potential participants were assessed from the registers, identified and screened for eligibility [35]. Of these, 69 participants were excluded from the study. This was mainly due to their sexual inactivity in the past 12 months [35]. This left 454 eligible participants.

### Data collection tools and procedures

The data for this study were collected from November 20, 2022 to April 30, 2023. A structured questionnaire (supplementary material 1) was the data collection tool used in this study. The tool was adapted from the previous studies conducted in Ethiopia [19,36–38] and Uganda [34,35]. The adaption entailed adding the hypothesized potential predictors for women’s fertility aspirations that were omitted in the previous studies such as the woman’s alcohol consumption, access or exposure to the FP information or counseling from the radio, knowledge about safer conception methods for YWLHIV, perceptions about safer conception methods, knowledge of contraceptive methods offered from the ART clinic facility and her current use of modern contraceptives. The questionnaire was validated for its item’s relevance, accuracy and comprehensiveness by a team comprising of midwives and obstetrician and gynecologists (the 2^nd^, 4^th^, 5^th^ and 6^th^ authors). They scored the questionnaire at a content validity index (CVI) of 97% which was way above the 70% lower limit of an acceptable CVI for a measuring tool. The questionnaire was translated to the local dialect (Langi) by a midwife (the 5^th^ author). The translator had a cultural background and training in health material translation from Langi to English language and vice versa.

Both the English and Langi versions of the adapted questionnaire were then pretested among 10% of the initial 423 sample size of the YWLHIV. The pretesting took place at the ART clinic of Lira University Hospital which was also located within the study area. The English and Langi versions of the questionnaire were both administered by one research assistant through face to face interview by facility. The feedback from the pre-testing were used to adjust and finalize the questionnaire for the actual study.

To collect data, face to face interviews were conducted by six trained nurse-midwife research assistants in English or Langi languages. This depended on the educational level and the language ability of the participants. The face to face interviews lasted for about one hour. Interviews were held in private spaces within the ART clinic facilities especially those free from an interruption by a third party.

### Study variables

#### Outcome variable.

The woman’s fertility desire was the outcome variable. Fertility desire was defined as the woman’s expressed desire to conceive and produce children despite her HIV status and past reproductive history. Accordingly, women were asked whether they desired to conceive and produce children despite their HIV positive status and past reproductive history. This data was later dichotomized into women with no fertility desires (coded as 0) and those with fertility desires (coded as 1). It was then used for the estimation of the fertility desire rate. The women who expressed the desire to have children were further asked about their desired number of children and also whether their male partners also desired to have children.

#### Explanatory variables.

The key explanatory variable was the integration of HIV care and FP services. This was measured by asking the women about the modern contraceptive methods they knew were being offered from the ART clinic facility where they usually obtain their HIV care. The responses generated from this item was categorized as those who did not know (coded 0) and those who knew one or more correct modern contraceptive methods (coded 1). The women were further asked whether they ever received any modern contraceptive method from the ART clinic facility. The women who reported ever receiving a modern contraceptive method from the ART clinic facility were coded 1 and those who did not receive were coded as 0. To confirm the methods received, the women were asked to specify the type of the method they received from the ART clinic facility. The methods were then categorized into three groups namely short-acting contraceptives (SAC), long-acting reversible contraceptives (LARC) and permanent methods.

The second key explanatory variable was the women’s knowledge and perceptions about safer conception methods for YWLHIV. This was measured by asking the women to state the safer conception methods that women living with HIV can use to conceive without transmitting the HIV infection to their male partners. Based on the responses to this item, women were categorized into those who did not know any safer conception method (coded 0) and those who knew one or more correct safer conception methods such as in-vitro fertilization or artificial insemination (coded 1). To assess perceptions, the women were asked what they thought were the benefits of using the safer conception methods for YWLHIV. Based on the responses to this item, women were categorized into those who did not perceive any benefit of the safer conception methods (coded 0) and those who perceived one or more correct benefits of safer conception methods such as preventing HIV transmission to the male partner (coded 1).

#### Confounding variables.

The key lifestyle related confounding variable assessed was the woman’s alcohol consumption. This variable was assessed by asking the women how much and how often they consumed alcohol. Based on the responses to this item, women were categorized into alcohol non-consumers (coded 0) and alcohol consumers (coded 1). Other lifestyle related factors assessed were the women’s drug use and tobacco smoking behaviors.

The other key confounding variable was the woman’s duration on ART. This variable was calculated from the year she was initiated on ART. Based on this, the women were categorized into those who have been on ART for a fewer year (≤5 years) and those who have been on ART for a longer year (6 + years). The additional ART – related variables assessed was the participant’s daily pill burden. This variable was assessed by asking how many medicines from the ART clinic usually was given to her to take on daily basis. Other potential confounding and explanatory variables assessed included the participant’s socio-demographic characteristics (particularly the age, having urban or rural residence, marital status, highest educational attainment and monthly income).

The women’s sexual and reproductive history was the other confounding variables assessed. This variable was assessed by asking the women about their age at sexual debut, gravidity, parity and the number of living children. The women were also asked about other sexual health indicators such as the HIV status of the male partner. This was aimed at determining the discordant status of their relationships. The other sexual and reproductive history asked were the woman’s male partner’s fertility desires and their current use of modern contraceptives. The women who reported a recent pregnancy within the past 12 months, were further asked whether their recent pregnancy was intended or unintended. The other sexual and reproductive histories assessed were the woman’s duration in the sexual relationships, the woman’s current pregnancy status, the woman’s current breastfeeding status, woman’s past miscarriage or abortion experiences and the woman’s sexual activity level.

Access to the community-based SRH resources related factors were as well assessed. Access to SRH resources were assessed by asking the women whether they have ever received FP information, education or counseling from the radio, television, posters or the billboards. They were also asked about their proximity to the health facilities in terms of distance. Other community-based SRH resource access related factors assessed included the woman’s awareness of the nearby providers of FP, private medical centres or clinics offering FP, drug shops or pharmacies offering FP, civil society organizations offering FP, community health workers also known as village health teams (VHTs) offering FP and the government health centres or hospitals offering FP.

### Data management and analysis

The data were double-entered in the EpiData 3.1 (EpiData Association, Odense, Denmark). It was then validated and exported to the IBM Statistical Package for Social Sciences (SPSS) version 26.0 (IBM Corp., Armonk, NY, USA). Descriptive statistics used to summarize the fertility desire rate and the participant’s characteristics were the frequency counts, percentages, mean, standard deviations (SD), median and the interquartile range (IQR). Associations between independent and outcome variables were assessed using the Chi-square or the Fisher’s Exact Tests. Factors showing significant associations at the bivariable analysis were first assessed for multi-collinearity before entry into the multivariable regression analysis. The multi-collinearity diagnostics analysis (shown in Table 1) indicate no severe multi-collinearity issues between the factors. This was because none of the factors had tolerance of ≤0.1, variance inflation factor of ≥5 and condition index of ≥30. And also no two factors simultaneously had variance proportions >0.5 in any dimension. The model appeared stable and therefore all the 14 factors were entered into the multivariable modeling. The results are shown in Table 3.

**Table 1:** Multi-collinearity diagnostic analysis between the factors associated with the women’s fertility desires.

Sn	Factor	Tolerance	Variance Inflation Factor (VIF).	Condition index	Two or more factors with variance proportions of >0.5 in the same dimension
1	Age	0.651	1.536	2.895	None
2	Marital status	0.689	1.451	3.229	None
3	Residence	0.641	1.561	4.008	None
4	Alcohol consumption.	0.769	1.300	4.155	None
5	Ever received FP method from the ART clinic facility	0.617	1.622	4.455	None
6	Years of sexual experience	0.619	1.617	4.670	None
7	Number of living children.	0.783	1.277	5.201	None
8	Unintended pregnancy experience	0.639	1.564	6.606	None
9	Years since HIV diagnosis	0.796	1.256	7.172	None
10	Knowledge of safer conception methods	0.475	2.107	8.149	None
11	Perceptions of safer conception methods	0.465	2.149	8.600	None
12	Ever received FP information, education or counseling from the radio	0.815	1.227	9.406	None
13	Knowledge of FP methods offered from the ART clinic facility	0.576	1.737	11.634	None
14	Currently using modern contraceptive method	0.695	1.440	18.745	None

ART is antiretroviral therapy and VIF is the variance inflation factor.

The goodness-of-fit statistics were assessed from the Poisson regression model. The model fit analysis indicated significant under dispersion with scaled deviance of 18.80 on 88 degrees of freedom (ratio = 0.21) and Pearson chi-square of 12.07 (ratio = 0.14). These suggested the model variance was substantially lower than the expected under standard Poisson assumptions. These were reflecting both the binary nature of the outcome and its high prevalence in the sample. Consequently, robust standard errors were employed for all inferential statistics to account for this variance misspecification.

While the dependent variable (fertility desires) had complete data, some of the covariates had missing values. Therefore, complete-case analysis was used to handle the missing data on the covariates. This unfortunately yielded a reduced analytic sample size, statistical power and potential for selection bias.

While complete-case analysis reduced the analytic sample size for the multivariable analysis from 423 to 103, the sensitivity checks supported the robustness of the findings. Attrition analysis revealed no significant differences between the included and excluded participants on the key variables particularly the woman’s HIV duration, marital status and number of living children. Furthermore, re-analysis using alternative handling of the missing covariates (in this case coding to the reference category) produced substantively similar results (associations). These checks confirmed that while the reduced sample size limits the statistical power, the observed associations were unlikely to be substantially biased by the missing data.

Independently associated factors were also identified using the modified Poisson regression with robust error variance**.** Each of the 14 factors (shown in Table 1) were first individually entered into the modified Poisson regression to generate the unadjusted measures of associations, the crude prevalence ratios (cPR). The above 14 factors (shown in Table 1) were later jointly entered into the modified Poisson regression to generate the adjusted measures of associations, the adjusted prevalence ratios (aPR). The measures of the associations were reported alongside their corresponding 95% confidence intervals. Statistical significance was set at p < 0.050.

### Ethical considerations

The ethical approval for the study was obtained from the Gulu University Research Ethics Committee (GUREC) under the approval number GUREC-2022–309. Informed consent was obtained from the participants aged 18 and above. For the participants aged 15–17 years, assent was obtained from their parents or legal guardians who were invited to the ART clinic facilities with their minors living with HIV. Outreaches were conducted to some participant’s homes with the guide from the community health workers affiliated to the ART clinic facilities. Both consent and assent were provided through written signatures or thumbprints. The six research assistants facilitated the consent process upon sampling and identification of the participants. The process entails ensuring the participants fully understood the study objectives, its procedures, risk, benefits and their rights to withdraw from the study at any time without any penalties before signing the informed consent or the assent forms.

The study adhered to the Declaration of Helsinki and other relevant ethical guidelines. Participants were compensated with 10,000 Ugandan shillings (approximately three U.S. dollars) for their time and participation in the study.

To ensure confidentiality during the active study phase, paper questionnaires were stored in a locked filing cabinet within the Principal Investigator’s (PI) secured office. They were accessible only to the research team. More so, all participant identifiers were removed during the data entry. This helped in creating a fully anonymized dataset. The digital dataset was stored on a password-protected, institutionally managed server with regular backups. All data were archived for five years’ post-publication. After which, paper records were shredded and the digital files were permanently deleted.

## Results

### Participant’s recruitment

**Fig 1:**
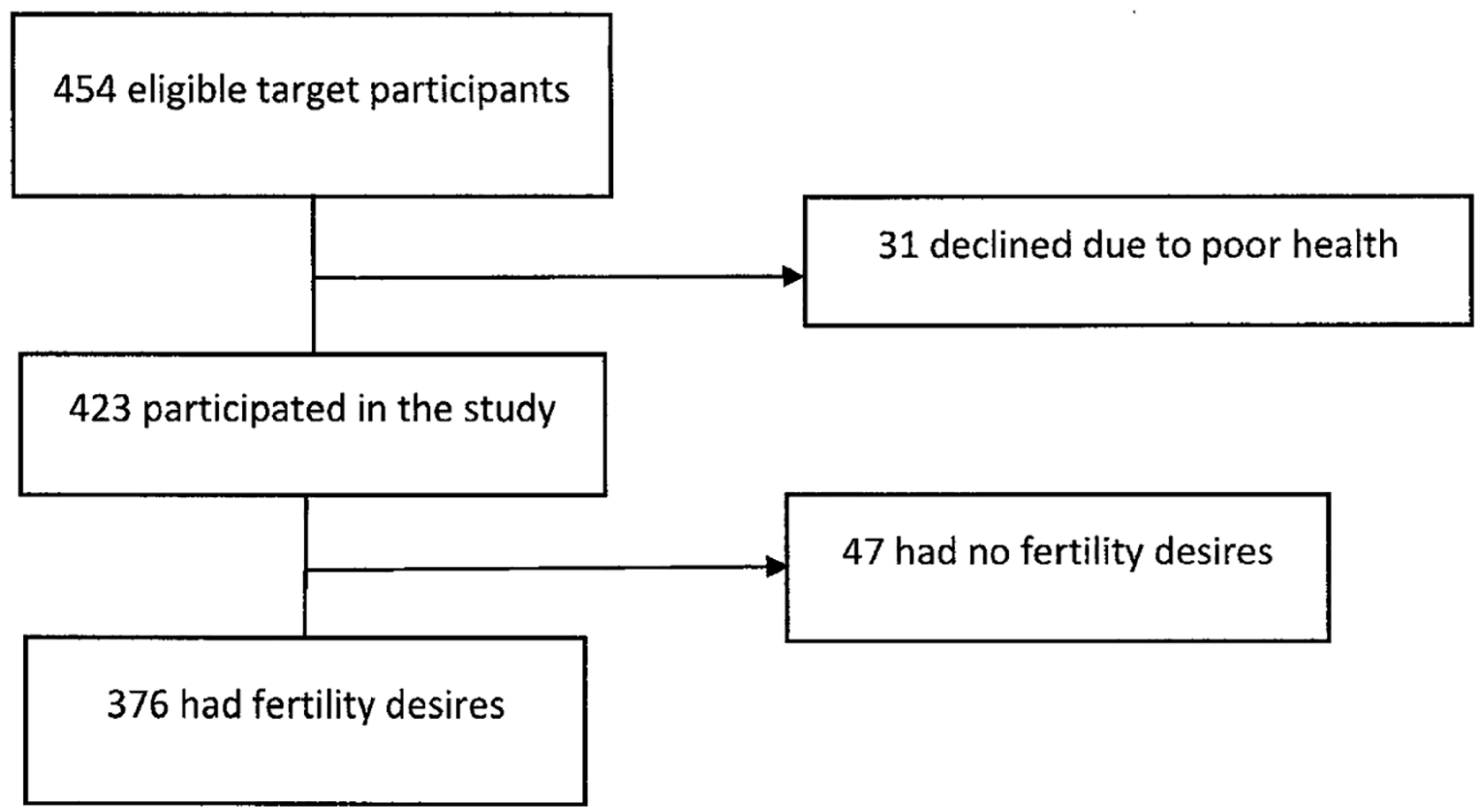
Flowchart showing the participant’s recruitment process.

As shown in Fig 1, the study successfully enrolled 423 YWLHIV. This achieved a 93.2% response rate from the target sample size of 454 YWLHIV. Of the 454 potential participants, 31 (6.8%) declined to join the study, primarily due to their poor health that prevented their involvement. There were no significant socio-demographic differences between the participants who joined the study and the non-participants. Additionally, no data were missing at random, as in person interviewers ensured all questionnaire items were completed.

### Participant characteristics

Table 2 summarizes the socio-demographic, SRH profiles of the YWLHIV. Participants were aged 15–24 years (median: 22; IQR: 20.0–24.0), with most being 20–24 years old. The majority of the participants were single, had no or only primary-level education and low-income earners. Most participants lived within 5 km of a health facility.

**Table 2:** Socio-demographic, sexual and reproductive health characteristics of the participants.

Factors.	F (%)
*Median age in years (IQR*	22 (IQR 20.0–24.0)
*age group in years* 15–19 20–24	99 (23.4) 324 (76.6)
*Religion* Christian Catholics Christian Other Denominations Islam	184 (43.5) 189 (44.7) 50 (11.8)
*Marital status* Single Married	220 (52.0) 203 (48.0)
*Educational attainment* No or Primary education Secondary education Tertiary education or higher	258 (61.0) 125 (29.5) 40 (9.5)
*monthly income in Uganda shillings (USD equivalent)* ≤87,600 (≤24) >87,600 (>24)	261 (61.7) 162 (38.3)
*residence* Rural area Semi-urban or urban	80 (18.9) 343 (81.1)
*alcohol use* Never Uses	372 (87.9) 51 (12.1)
*additive drug use* Never Uses addictive energy drinks and herbs	418 (98.8) 5 (1.2)
*pill burden* 1-2 types 3 or more types	412 (97.4) 11 (2.6)
*median age at sexual debut (IQR)*	17 (16.0-18.0)
*age at sexual debut* ≤15 years 16–19 years 20–24 years	105 (24.8) 259 (61.2) 59 (14.0)
*sexual debut period* Within 1 year Within 2–5 years 6 or more years	29 (6.9) 229 (54.1) 165 (39.0)
*Gravida* Nulligravida Gravida 1+	155 (36.6) 268 (63.4)
*Parity.* Nullipara Para 1+	200 (47.3) 223 (52.7)
*miscarriages and abortions* 0 1+	367 (86.8) 56 (13.2)
*woman’s number of living children* 0 1+	197 (46.6) 226 (53.4)
*recently gotten pregnant* No Yes	323 (76.4) 100 (23.6)
*Woman desires to bear children* No Yes	47 (11.1%) 376 (88.9%)
*Attending ART clinic with FP services* No Yes	207 (48.9%) 216 (51.1%)
*Ever received modern FP methods from the ART clinic* No Yes	372 (87.9%) 51 (12.1%)

USD is United States Dollars; F is frequency count; % is percentage; ART is antiretroviral therapy; FP is family planning; < is less than; > is greater than; ≤ is less or equals to and IQR is the interquartile range.

All the participants reported being sexually active. The mean recent sexual activity interval was 8.0 days (±15.7). Nearly half (48.0%) of the participants were married or cohabiting with male partners. The average relationship duration was 2.4 years (±2.4). Sexual initiation with their current partner occurred at a mean age of 19.0 (±2.6). Notably, 88.9% of the participants expressed a desire to have children.

### Fertility desires

As shown in Table 2, the study found that the majority 376 (88.9%, 95% CI 85.6% – 91.6%) of the YWLHIV in the study expressed a desire to have children. Only 47 (11.1%, 95% CI 8.4% – 14.4%) did not. The women’s desired number of children ranged from 1–10 with a median of 3 (IQR 2).

Similarly, out of the 423 YWLHIV who participated in this study, 312 (73.8%, 95% CI 69.4–77.9%) believed their male partners wanted children. Only 11 (2.6%, 95% CI 1.3–4.6%) said their male partners didn’t. Notably, 100 (23.6%, 95% CI 19.7–27.9%) were unsure of their male partner’s fertility desires. The male partner’s desired number of children ranged from 1–6 (IQR 1).

### Use of an integrated HIV and FP services

Of the 423 participants in the study, 51 (12.1%, 95% CI 9.0–15.2%) reported to have ever received FP services from the ART clinic facility where they usually obtain their HIV care. The majority 372 (87.9%, 95% CI 84.4–91.0%), did not (see Table 2). The utilization of the integrated HIV and FP services was significantly lower (30.1%) among the women with fertility desires compared to 48.9% among their counterpart without fertility desires (X^2^ 5.991, df 1, p = 0.014).

### Factors associated with women’s fertility desires from the bivariate analysis

Table 3 summarizes the socio-demographic, behavioral, reproductive and healthcare-related factors significantly associated with the YWLHIV’s desire to bear children at p < 0.050 from the bivariate analysis.

**Table 3:** Factors significantly associated with the women’s fertility desires from the bivariate analysis (N = 423).

Variable	Desire to bear children		X^2^	df	p-value
**No** **n (%)**	**Yes** **n (%)**
**Age** 15-19 20-24	4 (4.0) 43 (13.3)	95 (96.0) 281 (86.7)	5.641	1	0.018*
**Marital status** Single Married	34 (17.1) 13 (5.8)	165 (82.9) 211 (94.2)	12.462	1	<0.001*
**Residence** Rural Semi-urban or urban	17 (21.2) 30 (8.7)	63 (78.8) 313 (91.3)	9.042	1	0.003*
**Alcohol consumption** No Yes	35 (9.4) 12 (23.5)	337 (90.6) 39 (76.5)	7.682	1	0.006*
**Drug use** No Yes	44 (10.5) 3 (60.0)	374 (89.5) 2 (40.0)			0.011^F^*
**Attending ART clinic with FP services** No Yes	16 (7.7) 31 (14.4	191 (92.3) 185 (85.6)	4.047	1	0.044*
**Ever received FP method from the ART clinic** No Yes	24 (8.4) 23 (16.9)	263 (91.6) 113 (83.1)	5.991	1	0.014*
**Years of sexual experience** ≤5 6+	14 (6.8) 33 (15.3	192 (93.2) 183 (84.7)	6.831	1	0.009*
**Number of living children** **0** **1+**	12 (6.1) 35 (15.5)	185 (93.9) 191 (84.5)	8.480	1	0.004*
**Unintended pregnancy experience** No Yes	4 (5.9) 9 (25.7)	64 (94.1) 26 (74.3)			0.009^F*^
**Years since HIV diagnosis** ≤4 5+	12 (7.1) 35 (13.7)	156 (92.9) 220 (86.3)	4.443	1	0.035*
**Knowledge of safer conception methods** Do not know Correctly knows 1 + method	30 (15.2) 17 (7.6)	168 (84.8) 208 (92.4)	5.408	1	0.020*
**Perceptions about safer conception methods** No benefit perceived Correct benefits perceived	25 (15.4) 22 (8.4)	137 (84.6) 239 (91.6)	4.280	1	0.039*
**Received FP information from the radio** No Yes	10 (16.5) 27 (8.9)	101 (83.5) 275 (91.1)	4.298	1	0.038*
**Knowledge of contraceptive methods** Do not know Correctly knows 1 + methods	16 (7.7) 31 (14.4)	191 (92.3) 185 (85.6)	4.047	1	0.044*
**Currently using a contraceptive method** No Yes	26 (16.4) 21 (8.0)	133 (83.6) 243 (92.0)	6.261	1	0.012*

n is frequency count; % is percentage; X^2^ is Chi-square statistics; df is degree of freedom; p is the significance level; F is p-value obtained from the Fisher’s Exact test; ART is antiretroviral therapy and * is statistically significant p < 0.050.

Younger age women (15–19 years) were more likely (96.0%) to desire children than older women (20–24 years) whose fertility desire stood at 86.7% (p = 0.018). Single women were less likely (82.9%) to desire children compared to the married women whose fertility desire stood at 94.2% (p < 0.001). Urban women had a higher (91.3%) desire for children than rural women whose fertility desire stood at 78.8% (p = 0.003).

Women who consumed alcohol were less likely (76.5%) to desire children compared to the non-drinkers whose fertility desire stood at 90.6% (p = 0.006). Drug users had a sharply lower (40.0%) desire for children (p = 0.011).

Women diagnosed with HIV ≥ 5 years ago were less likely (86.3%) to desire children compared to those diagnosed ≤4 years whose fertility desire stood at 92.9% (p = 0.035). Women with no children were more likely (93.9%) to desire children than those with ≥1 children whose fertility desire stood at 84.5% (p = 0.004). Women who were not currently using any modern contraceptive method were less likely (83.6%) to desire children (p = 0.012).

Women attending ART clinic facilities without an integrated FP services were less likely (85.6%) to desire children compared to their counterparts (p = 0.044). The women who ever received FP method from the ART clinic facility were also less likely (83.1%) to desire children compared to their counterparts who did not (p = 0.014). Women who knew safer conception methods had higher (92.4%) fertility desire than their counterparts who did not know (p = 0.020). Similarly, those who perceived no benefits from the safer conception methods had lower (81.5%) desire to produce children compared to their counterparts who perceived benefits (p = 0.025). Women who received FP information, education or counseling from the radio had higher (91.1%) fertility desire than their counterparts who did not (p = 0.038).

### Other statistically significant associations between the factors from the bivariate analysis

The study further revealed that 87.5% of the single women were secondarily abstinent compared to just 39.5% of the sexually active married women (X^2^ 50.416, df 1, p < 0.001). Furthermore, single women used modern contraception methods about 20.8% less often than the married women (cPR 0.792, 95% CI 0.67–0.92, p = 0.003).

### Factors not associated with the woman’s fertility desires from the bivariate analysis

The study found numerous factors across four domains that showed no statistically significant associations with the women’s fertility desires (all with p-values ≥0.050). These statistically insignificant factors are not reflected in any of the tables but presented in the narratives below:

**Socio-demographics:** No significant associations were found between the woman’s fertility desires and the woman’s tribe (p = 0.781), the woman’s religion (p = 0.938), the woman’s educational level (p = 0.084), the woman’s employment status (p = 0.603) or the woman’s monthly income (p = 0.874).

**Health behaviors or access:** woman’s drug use (p = 0.067), woman’s awareness of any nearby providers of FP (p = 0.270), woman’s awareness of the private medical centres or clinics offering FP (p = 0.116), woman’s awareness of the drug shops or pharmacies offering FP (p = 1.000), woman’s awareness of civil society organizations or non-governmental organizations offering FP (p = 0.186), woman’s awareness of the village health teams offering FP (p = 1.000), woman’s awareness of the government health centres or hospitals offering FP (p = 0.416), woman’s ever receipt of FP information from the Television, posters or billboards (p = 0.652, 1.000 and 0.883 respectively), woman’s engagement in a recent unprotected sexual intercourse (p = 0.884), woman’s current use of any FP method including the traditional methods (p = 0.057), woman’s current use of the long-acting reversible contraceptive methods (LARC) (p = 0.722), woman’s current use of condoms (p = 0.316), woman’s previous failures to access modern contraceptive methods (p = 0.218) or the woman’s proximity to the health facility (p = 0.051) were not significantly associated factors to the woman’s fertility desires.

**Reproductive history:** Woman’s age at sexual debut (p = 0.845), woman’s duration in the current sexual relationships (p = 0.379), woman’s history of a recent pregnancy (p = 0.704), woman’s current pregnancy status (p = 0.058), woman’s current breastfeeding status (p = 0.050), woman’s history of miscarriages or abortions (p = 0.899) or woman’s sexual activity level (p = 0.259) showed no significant associations with the woman’s fertility desires.

**HIV-related factors:** woman’s mode of HIV acquisition (p = 0.147), woman’s method of HIV status discovery (p = 0.404), woman’s age at awareness of her HIV positive status (p = 0.140), woman’s years since HIV diagnosis (p = 0.051), woman’s HIV status disclosure to the current male partners (p = 0.168), male partner’s HIV status (p = 0.325), woman’s concordant or discordant status with the current male partners (p = 0.504), woman’s belief that the male partner desires fertility or children (p = 0.080), woman’s early ART initiation (p = 0.529), woman’s duration on ART (p = 0.360) or woman’s daily pill burden (p = 1.000) were not significantly associated with the woman’s fertility desires.

### Factors associated with woman’s fertility desires from the multivariable analysis

As shown in Table 4, the multivariable Poisson regression analysis revealed that several personal circumstances and healthcare system factors shape the reproductive aspirations of the YWLHIV.

**Table 4:** Factors associated with women’s fertility desires from the multivariable analysis (N = 103).

	Univariate analysis		Multivariable analysis	
Factors	cPR,95% CI	p-value	aPR,95% CI	p-Value
Being a teenager (compared to 20–24 year old)	1.10 (1.04-1.17)	0.001*	1.11 (0.87-1.42)	0.377
Being single (compared to married)	0.88 (0.82-0.94)	<0.001*	0.76 (0.62-0.94)	0.013*
Having a rural residence (compared to having semi-urban or urban residence)	0.86 (0.76-0.97)	0.015*	1.05 (0.82-1.35)	0.680
Not consuming alcohol (compared to being alcohol consumer)	1.18 (1.01-1.38)	0.033*	1.11 (0.87-1.42)	0.384
No history of receiving any FP methods from the ART clinic (compared to those who ever received FP from the ART clinic)	1.10 (1.01-1.19)	0.021*	1.23 (1.02-1.48)	0.025*
Having ≤5 years of sexual experience (compared to having 6 + years of sexual experience)	1.10 (1.02-1.17)	0.006*	1.04 (0.90-1.21)	0.550
Having no living children (compared to having 1 + children)	1.11 (1.04-1.18)	0.002*	1.17 (0.98-1.38)	0.068
No history of unintended pregnancy (compared to having a positive history of unintended pregnancy)	1.26 (1.03-1.55)	0.023*	1.17 (1.00-1.36)	0.040*
Having ≤4 years since HIV diagnosis (compared to having 5 + years since HIV diagnosis)	1.07 (1.00-1.14)	0.025*	1.04 (0.90-1.21)	0.550
Lack knowledge of the safer conception methods (compared to those with knowledge about the safer conception methods)	0.91 (0.85-0.98)	0.016*	1.03 (0.80-1.32)	0.780
Perceiving no benefits from the safer conception methods (compared to perceiving correct benefits of the safer conception methods)	0.92 (0.85-0.99)	0.039*	0.90 (0.68-1.18)	0.468
No history of receiving any FP information, education or counseling from the radio (compared to having received FP information from the radio)	0.91 (0.84-1.00)	0.049*	0.82 (0.66-1.03)	0.101
Having no knowledge of the FP methods offered from the ART clinic (compared to having correct knowledge of the FP methods offered from the ART clinic)	1.07 (1.00-1.15)	0.030*	0.82 (0.67-0.99)	0.045*
Being a non-current user of modern contraceptive method (compared to being a current user of the modern contraceptive method)	0.90 (0.84-0.98)	0.015*	0.77 (0.65-0.91)	0.003*

cPR is the crude prevalence ratio; aPR is the adjusted prevalence ratio; 95% CI is the 95% confidence interval; ≤ is less or equals to and * is statistically significant at p < 0.050.

We found three significant independent predictors associated with lower fertility desires among the YWLHIV. Firstly, single women had 23% lower fertility desire than their married counterparts (p = 0.013). Secondly, women attending ART clinic facilities without an integrated FP services had 18% lower fertility desire than their counters who were attending ART clinic facilities with an integrated FP services (p = 0.045). Thirdly, current modern contraceptive users had 23% lower fertility desire than their counterparts who were non-users (p = 0.003)

We also found two significant independent predictors associated with higher fertility desires among the YWLHIV at the multivariable analysis level. Firstly, women who ever received FP methods from the ART clinic facility had 24% higher fertility desire than their counterparts who did not receive any FP method from the ART clinic facility (p = 0.025). Lastly, women with history of intended pregnancies had 17% higher fertility desires than their counterparts with positive histories of unintended pregnancies (p = 0.040).

## Discussion

The study revealed that the majority (88.9%) of the YWLHIV expressed desires to have children. This demonstrates that being HIV positive does not eliminate childbearing aspirations. The high fertility desires among the YWLHIV found in this study conducted in northern Uganda challenges the common assumption that HIV-positive women might not want children. The 88.9% fertility aspiration rate found in this study conducted in northern Uganda is much higher than both the global rate of 42.1% [5] and the 6.7–58.0% rates reported from the previous studies conducted in Eastern, Central and Southwestern regions of Uganda [9,12,14,37,39], respectively. The high level of fertility aspirations reported in this study conducted in northern Uganda likely reflects the improved health service environment in northern Uganda. The environment characterized by universal access to ART/ PMTCT services and diminished rates of HIV-related stigma, discriminations or violence. This represents a significant shift from the context of the earlier research (2009–2019), where HIV-related stigma, discriminations or violence and limited ART/ PMTCT access were noted among the barriers to childbearing intentions among the YWLHIV.

Similarly, the 88.9% fertility desires found among the YWLHIV in this study conducted in northern Uganda is more than double the 15–40.8% rates reported from the other Eastern African countries like Kenya, Tanzania and Malawi [6,7,40–42]. It is also higher than the fertility aspiration rates reported from the Western and Central African countries such as Nigeria, Ethiopia, Democratic Republic of Congo (DRC) and Ivory Coast where the fertility aspiration rates ranged from as low as 7% to 80% [6,7,9,10,19,26,37,39,43,44]. The high fertility desires found among the YWLHIV in northern Uganda reflects improvements in the culture, relationships and medical care for the YWLHIV. Cultural norms deeply tie a woman’s value and identity to childbearing and motherhood. Similarly, marital stability often depends on a woman’s fulfilling her expected childbearing roles. Crucially, the universal access to ART and successful PMTCT programs have transformed HIV from being highly stigmatized and discriminated against disease condition into a stigma-free manageable chronic condition. The ART and PMTCT services allow the women to envision a future with healthy children. By and large, the high fertility desires among the YWLHIV in northern Uganda signals not only a claim to normalcy but also an urgent need for healthcare systems to integrate comprehensive reproductive services into the HIV care across SSA.

The study also revealed a significantly lower level of utilization of the integrated HIV and FP services among the YWLHIV with fertility desires (30.1%) compared to their counterparts without fertility desires (48.9%). The finding concurs with a previous study conducted in Ethiopia where the utilization of the integrated HIV and FP services was found at just above average 55.8% [23]. These studies concur in that they both found moderately low level of utilization of the integrated HIV and FP services. The moderately low level of utilization of the integrated HIV and FP services among the YWLHIV may be attributable to the inconsistent integrated delivery of the HIV and FP services. This translates into inconsistent FP education, counseling and method provisions from the ART clinic facilities. A previous study attributed the inconsistent integrated delivery of HIV and FP services to the multitude of health system constraints including the lack of policy guidance on integrated care, poor supervision or oversight, unclear service delivery guidelines, inadequate infrastructure and insufficient monitoring systems [45]. The moderate level of utilization of the integrated HIV and FP services needs further research into the associated factors. Nonetheless, these findings call for efforts to increase the uptake of the integrated HIV and FP services among the YWLHIV in SSA settings.

The study further revealed that single women exhibited 23% lower fertility desires compared to their married counterparts. This finding contradicts a global review and other previous studies conducted in Ethiopia and Nigeria where they found the opposite [5,19,38,46]. This substantial difference likely reflects the profound social and practical realities facing single women in the SSA context. Without the security of marriage, women may feel less inclined to pursue childbearing due to concerns about social stigma, financial instability or the challenges of single parenthood. These findings call for dedicated social support systems for single YWLHIV who desire to bear children.

Furthermore, the study found that women with higher fertility desires were significantly more likely to utilize the integrated HIV and FP services. Specifically, for every unit increase in fertility desire, there was an 82% higher likelihood of utilizing the integrated HIV and FP services (p = 0.045). This finding concurs with a previous study conducted in South Africa that demonstrated that integrating SRH and HIV care increased women’s fertility desires [21]. These findings suggest that the structure and comprehensiveness of healthcare services themselves may shape women’s reproductive aspirations. When HIV treatment facilities fail to incorporate reproductive health services, they inadvertently create barriers to accessing conception and contraception information, education or counseling.

The study also revealed that women with a positive history of a planned or intended pregnancies demonstrated 17% higher fertility desires than their counterparts with positive history of unplanned or unintended pregnancies (p = 0.040). This finding concurs with a previous study conducted in southwestern Uganda [9]. They imply that previously successful planned or intended conception or pregnancy can encourage or motivate the YWLHIV to develop more childbearing aspirations to meet their reproductive goals. The finding also implies that an integrated HIV and FP program should routinely screen women for their past reproductive history. The program should also offer conception and contraceptive information, education, counseling and methods to the women with positive histories of planned conception or pregnancy to reduce the risk of unintended pregnancy and the associated consequences.

Most strikingly, current users of modern contraceptive methods demonstrated 23% lower fertility desires than their non-user counterparts (p = 0.003). This finding concurs with a previous study conducted in southwestern Uganda, DRC and northcentral Nigeria where they also found that contraceptive use were associated with reduced odds of pregnancy intention [11,36,47]. This inverse association between current use of modern contraceptives and fertility desires reflect rational decision-making where women actively using contraceptives are intentionally preventing pregnancy because they currently do not desire to have children. These findings imply that fertility desires are shaped by access to birth control methods. For clinicians and policymakers, these findings emphasize the need for comprehensive patient-centered approaches that address both the medical and social dimensions of SRH care for the YWLHIV.

### Study strengths

This study boast of several important strengths. Firstly, it used a statistically determined sample size that was large enough to produce generalizable results. Secondly, it examined a comprehensive set of predictor variables that previous studies had linked to women’s fertility desires and other unexamined factors. These included the socio-demographic characteristics, SRH factors and the HIV-specific and ART-related variables. The inclusion of these multitude of factors assures the practical relevance of the research findings.

### Study limitations

This research had some limitations. While our sample size was adequate for estimating the overall prevalence of fertility desire with good precision, it was not formally powered for detecting all associations between the covariates and the outcome (fertility desire). Thus, non-significant findings in subgroup analyses should not be interpreted as evidence of no effect. Similarly, the significant findings require confirmation in larger, hypothesis-driven studies.

The study did not collect data on the male partner’s attendance of the HIV care and on the women’s exposure to PMTCT information, education, counseling, knowledge or perceptions which were shown to have significant associations with YWLHIV’s fertility desires in previous studies conducted in Ethiopia [25,46]. Future studies should include validated survey modules on PMTCT and male partner’s engagement in the HIV care as standard covariates, given their established significant associations with the YWLHIV’s fertility aspirations.

Furthermore, social desirability bias may have influenced the women’s expressed fertility desires, given the sensitive nature of the reproductive decisions and potential social pressures related to factors like age and marital status. While we attempted to mitigate this bias through supplementary questions about their reproductive history (including gravidity, parity, number of living children and reproductive goal or desired family size), some reporting inaccuracies may remain.

More so, the positive association between the women’s fertility desires and the utilization of the integrated HIV and FP services should be interpreted with caution. This is because of the limitations associated with the self-reported nature of the data on HIV and FP service integrations and the cross-sectional design of the study. The cross-sectional design and reliance on self-reported data prevented the determination of whether higher fertility desires lead to seeking integrated services or whether exposure to the integrated HIV and FP services influences the stated fertility desires. To minimize this limitations, we employed rigorous statistical control for a wide range of potential confounders (e.g., age, parity, marital status, woman’s beliefs about the male partner’s fertility desire, woman’s socioeconomic status, knowledge of FP methods available and offered from the ART clinic facility, and distance to the ART clinic facility). These helped to reduce the likelihood that the observed association was spurious.

Another study limitation was the missing data on the covariates. This reduced the sample size for the multivariable analysis. While complete-case analysis preserved the internal consistency, it may have introduced selection bias and limited the generalizability of the findings. Findings should therefore be interpreted with caution as it requires replication with more complete cases.

Another limitation was our inability to directly assess male partners’ fertility aspirations. While we collected the women’s beliefs about their male partners’ childbearing desires, this indirect measure may not have fully captured the male partner’s actual perspectives. The absence of paired couple data remains a constraint for understanding the dyadic fertility decision-making in this context and should be addressed in future studies.

## Conclusions

This study demonstrates that the YWLHIV in Northern Uganda maintain strong childbearing aspirations and reveals a fertility desire rate significantly higher than the global and regional averages. Despite the higher fertility aspirations, the uptake of the integrated HIV and FP services remained low. The determinants of low or higher utilization of the integrated HIV and FP services warrants further investigations. The key independent factors associated with the women’s fertility intentions include her marital status (with single women showing lower fertility desires), access to the integrated FP services (with lower fertility desires among women attending the non-integrated clinics), prior pregnancy planning (with higher fertility desires among women with positive history of planned or intended pregnancies) and contraceptive use (with lower fertility desires among modern method users). These findings underscore the complex interplay of social, healthcare and personal factors in shaping the reproductive decisions.

The study also highlights the necessity for integrating FP services within the HIV care programs to support the YWLHIV in achieving their fertility goals safely. Policymakers and clinicians should prioritize addressing marital expectations and contraceptive access issues. Despite the limitations like potential social desirability bias and lack of male partner’s perspectives, this study provides critical insights for improving SRH care for the YWLHIV. Improving SRH care for the YWLHIV will ensure their reproductive rights are upheld alongside the HIV disease management. Future studies should include male partners to better understand the dyadic decision-making. It should also explore longitudinal fertility outcomes of a cohort of this population. More so, future studies on the factors influencing the utilization of the integrated HIV and FP services among the YWLHIV in other SSA settings like Uganda are also desirable.

## Abbrevations:

ART: Antiretroviral therapy
DRC: Democratic Republic of Congo
FP: Family planning
IQR: Interquartile range
LARC: Long-acting and reversible contraceptives
PLHIV: People living with HIV
SAC: Short-acting contraceptives
SPSS: Statistical package for social sciences;
SRH: Sexual and reproductive health
SSA: sub-Saharan Africa
YWLHIV: Young women living with HIV
