# Supplementary material for: Fertility desires and utilization of an integrated HIV care and family planning services among young women living with HIV in semi-rural Uganda: A cross sectional study
Source: PLoS One. 2026 Jul 24;21(7):e0349072. doi: 10.1371/journal.pone.0349072 (PMC13399477; doi:10.1371/journal.pone.0349072)
Supplement: Attachment — Submitted filename: RESPONSES TO REVIEWERS.pdf [file pone.0349072.s001.pdf]

RESPONSE TO THE REVIEWERS

**COMMENTS**	**RESPONSES**
**Editor's Comments**	
1. Please ensure that your manuscript meets PLOS ONE's style requirements, including those for file naming.<br>The PLOS ONE style templates can be found at https://journals.plos.org/plosone/s/file?id=wjVg/PLOSOne_formatting_sample_main_body.pdf and https://journals.plos.org/plosone/s/file?id=ba62/PLOSOne_formatting_sample_title_authors_affiliations.pdf	The manuscript meets PLOS ONE's style requirements.
2. Your ethics statement should only appear in the Methods section of your manuscript. If your ethics statement is written in any section besides the Methods, please move it to the Methods section and delete it from any other section. Please ensure that your ethics statement is included in your manuscript, as the ethics statement entered into the online submission form will not be published alongside your manuscript.	The ethics statement is only on the methods section on page 14 in lines 388-409.
3. If the reviewer comments include a recommendation to cite specific previously published works, please review and evaluate these publications to determine whether they are relevant and should be cited. There is no requirement to cite these works unless the editor has indicated otherwise.	Thank you for the guidance.
Introduction<br>Address coherency issues in this section<br>Lines 81-84 you wrote<br>*In countries like the United Kingdom (UK), a person living with HIV (PLHIV) is not permitted to donate their eggs or sperm to another person or couple undergoing fertility treatment including their own friends and family (4).*<br>Does not match with statement before, as context is different – delete or use example from a similar context.	We have used example from South Africa which is a similar sub-Saharan African context to Uganda.
Lines 102- opening statement for paragraph, you wrote<br>*Despite the availability of ART and FP services and their reproductive health benefits for PLHIV, little is known about the fertility aspirations of the YWLHIV in SSA settings such as Uganda, their utilization of an integrated HIV care and FP services, and the factors influencing their reproductive decisions is not appropriate as focus in on reproductive desires and factors*<br>Rephrase this paragraph to improve clarity and coherency. Consider splitting into two statements to address this	*In line 100 – 102, we have improved the clarity and coherency of paragraph by splitting the long unclear sentences into two shorter sentences.*
Discussion<br>Amend sentence line 488. *The study revealed that an overwhelming majority (88.9%) of YWLHIV expressed a desire*	Under discussion in line 561, we have deleted the word "overwhelming".

The use of 'overwhelming' makes statement sound informal and subjective.<br>Rather delete the word, and write e.g., *The study revealed that majority (88.9%) of YWLHIV expressed a desire…*	
Discussion<br>Work on critical analysis in your discussion section, for instance:<br>line 525 -527 you wrote<br>*The study also revealed that women with a positive history of a planned or intended pregnancy demonstrated 17% higher fertility desires (p=0.040). This finding concurs with a previous study conducted in southwestern Uganda (14). This suggest that access to conception or pregnancy planning may encourage childbearing intentions*<br>Paragraph is too short. Consider the last statement in this paragraph and develop this further for critical analysis, for instance, what does this imply for YWLHIV, or why is this important to YWLHIV, what is the implication for integrated ART and FP services	In line 620-629, we have added critical analysis and implication of the finding that women with a positive history of planned or intended pregnancy were more likely to have higher fertility desires than their counterparts.
Discussion<br>Minor<br>Amend typo errors by deleting full stop in all your headings	We have removed all the full stops in all headings across the entire manuscript.
Discussion<br>*Line 533 and 538 you used the word 'don't'*<br>Avoid contraction like 'don't in academic writing, change to 'do not' for a more formal writing	We have changed the word don't with do not throughout the manuscript.
**REVIEWER #3:**	
1.General comments: very interesting study. It would seem there is a dearth of literature in this area.	Thank you
2. Introduction Section: Too long and winding, comprehension is difficult.	We have shortened the introduction section and made it clearer by introducing a paragraph on fertility desires.
3. Methodology Section:<br>a. Outcome Variable section of the article:<br>i. Authors have defined the term "fertility desires" in the Outcome Variable section of the Methodology. However, it will be better to being the early into the Introduction for better comprehension of the paper.	In lines 78-91, we have defined fertility desires early in the introduction section.
3. Methodology Section:<br>a. Outcome Variable section of the article:<br>ii. Under the Outcome Variable section, the sentence "Accordingly, women whether they desire to conceive and produce more children despite their HIV positive status and	In line 282-283 under methodology, we revised the sentence to "Accordingly, women were asked whether they desire to conceive and produce more children despite their

reproductive history.” is not complete and should be re-phrased.	HIV positive status and past reproductive history”.
3. Methodology Section:<br>a. Outcome Variable section of the article:<br>iii. This sentence needs to be rephrased and English improved for better comprehension. “The women who expressed desire to have children where further asked about their desired number of children and also whether they male partner also desired to have children.”	In line 285-287 under the methodology, we revised the sentence to “The women who expressed the desire to have children were further asked about their desired number of children and also whether their male partners desired to have children.”
Methodology Section:<br>b. Explanatory Variable section:<br>i. Authors should use “did not” instead of “didn’t” on line 255.	In line 296 under explanatory variable, we changed the word “didn’t” to “did not.”
4. Results Section:<br>a. Table 2, authors should replace “address” with “residency” or perhaps “location”.	In table 2, we have replaced “address” with “residence”.
Results Section:<br>b. Authors are recommended to include the fertility desires and Integration of HIV and FP services into the Table 2 to improve comprehension.	We added the results for fertility desires and integration of HIV and FP services into Table 2.<br><br>Additionally, in lines 444-461, we presented the results for fertility desires and the integrated utilization of HIV and FP services as separate sections since they are among the key variables of the study. These separate sections allowed us to present more details such as the 95% confidence interval and range.
Results Section:<br>i. In general, one is not sure which narrative is for which table. After reading I realized that if only significant variables were included in Table 3 while non-significant variables were described below. However, there is an inconsistent arrangement in the narratives and tables, making it difficult to understand the Results section.	In lines 465-466 and 496, we have made it clear that Table 3 shows the factors significantly associated with the woman’s fertility desires from the bivariate analysis and therefore the narratives in lines 463-487 are for the results in Table 3.<br>In line 506-507, we indicated that the insignificant factors are not shown in any table but presented in the narrative texts.
Results Section:<br>c. Authors should desist using acronyms such as “vs.” in the write up.	In lines 473 and 477 and also in table 4, we have replaced the acronym “vs” with “compared to”.

Results Section:<br>d. The following variables are not in Table 3.<br>i. “Women attending ART clinic facilities without FP services were less likely to desire children (85.6%) (p=0.044)”.	In table 3, we have added the variable women attending ART clinic with integrated FP services plus the corresponding association with fertility desires measures.
Results Section:<br>d. The following variables are not in Table 3.<br>ii. “Drug users had a sharply lower desire for children (40.0%) (p=0.011).”	We have added data on drug use and its association with fertility desires into Table 3.
Results Section:<br>e. In general, authors should ensure that the narratives of tables actually reflect what is in the tables.	We have ensured the textual narratives of tables actually reflect data in the tables.
5. Discussion:<br>a. The authors should explain how does “The study finding concurs with previous conducted in Ethiopia where the utilization of an integrated HIV and FP services was found at 55.8% ..”	In line 590-597 under discussion, we explained how the finding from northern Uganda concurs with a previous study conducted in Ethiopia where the integrated utilization of HIV and FP services was found at 55.8% in that both were moderately low level of utilization.
Discussion<br>b. The authors should offer possible reasons why their findings concur or do not align with literature with supporting references.	In lines 590-597 under discussion, we have provided the possible reasons why the findings concur in terms of both having a moderately low level of integrated utilization of HIV care and FP services with supporting references.
Discussion<br>c. Authors should replace “don’t” with “do not”	We have replaced “don’t” with “do not” throughout the manuscript.
6. Study Strength<br>a. The authors check the language	In line 646 - 651 under study strengths, we have corrected the typos that were in the sentences.
7. Conclusion<br>a. Authors mention semi-rural in the conclusion but assessed for YWLHIV in urban, semi-urban and rural areas in the results section. What is the reason for the discrepancy?	In lines 692 under conclusion, we have replaced semi-rural with northern Uganda. It was a typo.
**REVIEWER #4**	
1. Study Design and Methodology:<br>1.a). The cross-sectional design is appropriate for the stated objectives. However, details on sampling procedures are limited. The selection criteria for ART clinics, proportional allocation of participants, and handling of non-respondents should be clearly described to assess representativeness.	In lines 210 to 252 under sampling and participant recruitment section, we provided the details on the selection criteria for the ART clinic facilities, sampling procedures and handling of non-respondents.

Study Design and Methodology:<br>1.b). Clarify how the questionnaire was validated, translated, and pre-tested.	In lines 261-267, we explained how the questionnaire was validated.<br><br>More so, in lines 268-272, we explained how the questionnaire was pre-tested.
Study Design and Methodology:<br>Since fertility desire is the central outcome, the exact question wording should appear in the Supplementary Material.	The supplementary material (data collection tool) has items H1-H6 to measure the fertility desire as the main outcome variable.
Study Design and Methodology:<br>1.c). The section on ethics should be expanded to include institutional review board approval reference, consent procedures (particularly for minors), and data confidentiality measures.	In lines 392-394, we added details on consent procedure particularly for minors.<br><br>Furthermore, in lines 403-409, we added the data confidentiality measures.
2. Data Management and Statistical Analysis:<br>2.a). The use of modified Poisson regression is methodologically appropriate; however, reporting is inconsistent. Both crude and adjusted prevalence ratios (PRs) with 95% confidence intervals should be presented for all predictors in a single comprehensive table.	In table 4, we have presented the crude and adjusted prevalence ratios (PRs) with their corresponding 95% CIs for all the associated factors in a single comprehensive table 4.
Data Management and Statistical Analysis:<br>2.b). Clarify discrepancies between text and tables (e.g., Table 4 shows N = 103 whereas the overall sample is n = 423). Explain whether this refers to a subset or correct it if erroneous.	In line 557 above Table 4, we maintained the N=103 explained in lines 373-374 under data management analysis that complete-case analysis reduced the analytic sample size for the multivariate analysis from 423 to 103.
Data Management and Statistical Analysis:<br>2.c). The discussion of multi-collinearity diagnostics is inaccurate. Acceptable thresholds are typically tolerance ≤ 0.1 and VIF ≥ 5. Provide the actual VIF values per variable and correct the explanation accordingly.	In line 354 – 355 under multi-collinearity analysis, we indicated that the acceptable thresholds are typically tolerance ≤ 0.1 and VIF ≥ 5. Accordingly, in table 1, we have provided the actual tolerance and actual VIF values per variable.
Data Management and Statistical Analysis:<br>2.d). Differentiate clearly between bivariate and multivariable findings.	In lines 463, table 3, lines 496, 503, 540, 556 and table 4, we have clearly specified the bivariate and multivariate analysis findings.
Data Management and Statistical Analysis:<br>Avoid interpreting non-significant results as meaningful associations.	In lines 503-538, we have maintained the insignificant results to enable the readers see the factors with insignificant associations with fertility

	desires that were excluded from the multivariate analysis.
3. Results and Interpretation:<br>3.a). The high fertility desire (88.9%) is striking and should be contextualized. Discuss how cultural expectations, marital norms, and optimism about ART access might influence reproductive intentions among YWLHIV.	In lines 568-572 under discussion section, we discussed the cultural expectations, marital norms and optimisms about ART access that might have influenced the reproductive intentions among YWLHIV.
3.b). Interpret differences in FP utilization between those with and without fertility desires cautiously, acknowledging self-report and cross-sectional limitations.	In lines 668 – 678 under the study limitations, we acknowledged the need to cautiously interpret the association between the YWLHIV's fertility desires and their FP utilization from the ART clinic, acknowledging the limitations from the self-reported nature of data and the cross-sectional study design.
3.c). Include a concise statement on model fit, handling of missing data, and sensitivity checks.	In lines 362-380 under the data management and analysis, we have provided concise statements on model fit, handling of missing data and sensitivity checks.<br><br>More so in lines 680-684 under the study limitations, we have acknowledged the reduced sample size, statistical power and possible selection bias that may have arisen from the missing data on the covariates.
4. Discussion and Conclusions:<br>4.a). The discussion currently summarizes results but lacks analytical depth. Strengthen this section by comparing findings with similar studies from other East African countries (e.g., Kenya, Malawi, Tanzania) and discussing contextual determinants such as gender roles, stigma, and health system barriers.	In lines 574-586, we have compared the current study findings from northern Uganda with similar studies from other East African countries (e.g., Kenya and Tanzania). Malawi is not regarded as an East African country.
Discussion and conclusion<br>4.b). Revise all causal language to reflect associations only (e.g., "factors associated with" rather than "predictors of").	We have revised all the causal languages to reflect associations only for example we replaced predictors with factors associated with throughout the manuscript.
5. Presentation and Writing Quality:<br>5.a). The manuscript is generally clear but requires language editing for grammar, tense, and precision.	We have edited the entire manuscript for clarity of the language, grammar, tense and precision.

Presentation and writing quality<br>5.b). Use the term "integrated HIV and FP services" consistently throughout.	We have consistently used the phrase integrated HIV and FP services throughout the manuscript.
Presentation and writing quality<br>5.c). Ensure uniform presentation of p-values, percentages, and confidence intervals across text and tables.	We have presented p-values in 3-places of decimals, percentages in 1-place of decimal and confidence intervals in 2-places of decimals across text and tables.
Presentation and writing quality<br>5.d). Add a Data Availability Statement and deposit anonymized data and analysis code in a public repository (e.g., Zenodo, OSF, or Figshare), following PLOS ONE's open data policy.	In lines 892-894, we have added a data availability statement and included a statement that anonymized data will be deposited in a public repository for open data policy.
