# Supplementary material for: Fertility desires and utilization of an integrated HIV care and family planning services among young women living with HIV in semi-rural Uganda: A cross sectional study
Source: PLoS One. 2026 Jul 24;21(7):e0349072. doi: 10.1371/journal.pone.0349072 (PMC13399477; doi:10.1371/journal.pone.0349072)
Supplement: Attachment — Submitted filename: RESPONSES TO REVIEWERS 3.pdf [file pone.0349072.s002.pdf]

RESPONSE TO THE REVIEWERS 3

COMMENTS	RESPONSES
**Editor's Comments**	
1. If the reviewer comments include a recommendation to cite specific previously published works, please review and evaluate these publications to determine whether they are relevant and should be cited. There is no requirement to cite these works unless the editor has indicated otherwise.	There were no reviewer's recommendations to cite specific previously published works.
2. Please review your reference list to ensure that it is complete and correct. If you have cited papers that have been retracted, please include the rationale for doing so in the manuscript text, or remove these references and replace them with relevant current references. Any changes to the reference list should be mentioned in the rebuttal letter that accompanies your revised manuscript. If you need to cite a retracted article, indicate the article's retracted status in the References list and also include a citation and full reference for the retraction notice.	I conducted a systematic check by searching for each article title and its authors in the Retraction Watch Database, the most comprehensive and reliable source for this information. For the 46 specific titles you provided, I found no corresponding retraction notices or entries indicating they have been withdrawn.<br><br>I also checked the publisher's website for updated editions or corrigenda for reports and data from organizations like references 1, 2 and 17 and found no updated reports as yet.
3. Additional Editor Comments:<br>- Please note that PLOS ONE does not copyedit manuscripts, so the language in submitted articles must be clear, correct and unambiguous. Please work to improve the quality of the writing throughout your manuscript. We recommend enlisting the help of a professional copyediting service.	We enlisted the help of a professional copyediting service, and ensured the language in the submitted articles are clear, correct and unambiguous.
**Reviewer's Comments**	
Reviewer #4: The authors have adequately addressed the concerns raised in the previous review. The study design and methodology are now clearly described, including sampling procedures, participant recruitment, proportional allocation, and management of non-response. The development of the questionnaire has been clarified, with details on validation, translation, and pre-testing, and the exact wording of the fertility desire outcome has been provided in the Supplementary Material. Ethical approval, consent procedures (including for minors), and data confidentiality measures are appropriately documented.<br><br>The statistical analysis is now clearly and consistently reported. The use of modified Poisson regression is appropriate, and both crude and adjusted prevalence ratios with 95% confidence intervals are presented in a single table. Earlier inconsistencies in sample size reporting have been resolved. The explanation of multicollinearity has been corrected using accepted thresholds, with relevant diagnostic values reported. The authors clearly distinguish between bivariate and multivariable results and avoid causal language, which is appropriate given the cross-sectional design.<br><br>The Results and Discussion have been strengthened by	Thank you.

better contextualization of the high fertility desire among young women living with HIV, including discussion of sociocultural norms, marital expectations, and access to ART, and by comparison with findings from other East African settings. Policy and programmatic implications are clearly stated. The manuscript now meets PLOS ONE requirements for clarity, reporting, and data availability.	
