# Supplementary material for: Fertility desires and utilization of an integrated HIV care and family planning services among young women living with HIV in semi-rural Uganda: A cross sectional study
Source: PLoS One. 2026 Jul 24;21(7):e0349072. doi: 10.1371/journal.pone.0349072 (PMC13399477; doi:10.1371/journal.pone.0349072)
Supplement: Attachment — Submitted filename: RESPONSE TO REVIEWERS 5.pdf [file pone.0349072.s004.pdf]

RESPONSE TO REVIEWERS

COMMENTS	RESPONSE
If the reviewer comments include a recommendation to cite specific previously published works, please review and evaluate these publications to determine whether they are relevant and should be cited. There is no requirement to cite these works unless the editor has indicated otherwise.	Not applicable.
Please review your reference list to ensure that it is complete and correct. If you have cited papers that have been retracted, please include the rationale for doing so in the manuscript text, or remove these references and replace them with relevant current references. Any changes to the reference list should be mentioned in the rebuttal letter that accompanies your revised manuscript. If you need to cite a retracted article, indicate the article's retracted status in the References list and also include a citation and full reference for the retraction notice.	The reference list is reviewed, complete and correct
1) Line 441 states that the majority of participants were married, but the results in Table 2 indicate that most were single (52% single vs 48% married).	In line 441, we have corrected that the majority of the participants were single.
2) We note that on line 457 (and others), the frequency and 95% CI is not presented in a standard format. The correct format is given on line 469 [i.e. 51 (12.1%, 95% CI 9.0 – 15.2%)]. Please ensure that this is how the frequency and 95% CI is stated throughout the manuscript, rather than [376 (88.9%) with a 95% CI of 85.6% –91.6%].	In lines 457 – 465, we have used the correct format to presented the frequency count, percent and 95% CI.
3) There are other copyediting errors/inconsistencies throughout the manuscript. Please ensure a thorough revision, or enlist the help of a professional copyeditor, to correct these and improve the quality of the writing.	We have enlisted the help of a professional copyeditor to correct all the copyediting errors/ inconsistencies throughout the manuscript.
Reviewer #5: Without formal hypothesis statements and a power consideration the investigators used the Kish Leslie formula for sample size determination. The assumptions included a 50% prevalence, 1.96 standard deviation corresponding with 95% confidence level from the standard normal distribution curve and a 0.05 margin of error. This gave the minimum required sample size of YWLHIV. This initial sample size was further	We thank the reviewer for this important methodological observation. We acknowledge that our initial sample size calculation, based on the Kish Leslie formula, was indeed designed to estimate a prevalence (descriptive objective) with a desired precision (5%), rather than to test a specific a priori hypothesis for inferential comparisons.

adjusted for a 10% non-response rate resulting into a final target sample size of 423 YWLHIV.	We recognize that, ideally, sample size should be determined by power calculations tied to the formal hypothesis statements for the primary analytical comparisons. However, given the exploratory nature of this study and the lack of prior published data on key associations within this specific population of YWLHIV in northern Uganda setting, we prioritized obtaining a sufficiently large and precise descriptive estimate. The final achieved sample of 423 YWLHIV was a pragmatic balance between statistical precision and logistical feasibility.<br><br>To address concerns regarding analytical power, we conducted a post-hoc sensitivity analysis to evaluate the minimum detectable effect sizes for our secondary/ comparative analyses at 80% power and α=0.05, with our achieved sample of 423 participants:<br><br>• For estimating a single proportion, our 95% confidence intervals around any observed prevalence have a maximum width of approximately ±4.8%, confirming that our precision objective was met.<br>• For comparing two independent subgroups of roughly equal size, our sample had 80% power to detect a difference of approximately 12–13 percentage points between the groups.<br>• For a logistic regression model (if applicable), our sample size of 423 comfortably satisfies the standard 'events-per-variable' rule of thumb (at least 10–15 events per predictor), allowing us to fit a stable multivariable model."<br><br>We have now clearly stated this as a sample size determination in the revised manuscript (see lines 217-233).<br><br>Therefore, while we agree that formal a priori power calculations for each hypothesis would have strengthened the study design, we are confident that the final sample size of 423 provides adequate precision for our primary descriptive aim and sufficient sensitivity to

	detect clinically meaningful differences in our exploratory analyses.<br><br>We have now clearly stated this as a limitation in the revised manuscript (see lines 710-714).
The structured questionnaire and explanatory and confounding effects were reasonably considered, The data management and analysis were targeted for mostly a descriptive type of presentation with some inferential considerations..<br>Descriptive statistics, parametric and non-parametric, were used to summarize the fertility desire rate and the participant's characteristics were the frequency counts, percentages, mean, standard deviations (SD), median and the interquartile range (IQR). Associations between independent and outcome variables were assessed using the Chi-square or the Fisher's Exact Tests. Factors showing significant associations at the bivariate analysis were first assessed for multi-collinearity before entry into a multivariable regression analysis. The analysis was simple overall and the progression from bivariate to multiple regression with the appropriate strategies including the appropriate models applied were easily followed. The tables were well formatted and easily presented. The conclusions followed from the analyses performed.<br>The progression of the 3 revisions appeared reasonable as per the tolerances of the statistical boundary cutoffs as per the previous reviewers. The investigators responded appropriately. The added note from the third reviewer that the statistical analysis is now clearly and consistently reported is certainly correct. The added use of modified Poisson regression is appropriate, and both crude and adjusted prevalence ratios with 95% confidence intervals are presented in an appropriate format as noted.	We are deeply grateful to the reviewer for the thorough and positive evaluation of our statistical methods and analysis.<br><br>We greatly appreciate the time and expertise invested in reviewing the progression of our revisions and the final analytical approach.<br><br>We are particularly encouraged by the reviewer's confirmation that:<br>• The structured questionnaire and consideration of explanatory and confounding effects were reasonable;<br>• The descriptive and inferential analyses, including the use of parametric and non-parametric methods, were appropriate;<br>• The progression from bivariate analysis to multivariable regression, with appropriate checks for multi-collinearity and the use of modified Poisson regression, was clearly presented and methodologically sound;<br>• The presentation of both crude and adjusted prevalence ratios with 95% confidence intervals is appropriate; and<br>• The conclusions are consistent with the analyses performed.<br><br>We are pleased that the statistical reporting now meets the standards expected by the reviewer and the journal. We have carefully reviewed the manuscript once more to ensure that all statistical terminology is consistent throughout, and we have made minor editorial refinements to enhance clarity where possible (these are highlighted in the revised version).<br><br>We thank the reviewer again for their constructive engagement throughout this process, which has significantly strengthened the quality of our manuscript.
