# Supplementary material for: Fertility desires and utilization of an integrated HIV care and family planning services among young women living with HIV in semi-rural Uganda: A cross sectional study
Source: PLoS One. 2026 Jul 24;21(7):e0349072. doi: 10.1371/journal.pone.0349072 (PMC13399477; doi:10.1371/journal.pone.0349072)
Supplement: Peer review history

# Peer review: Decision Letter 0

**DOI:** 10.1371/journal.pone.0349072.r001

**Authors:** Hannah Mafo Degge

17 Nov 2025

PONE-D-25-45727

Fertility desires and utilization of an integrated HIV care and family planning services among young women living with HIV in semi-rural Uganda. A cross sectional study.

PLOS ONE

Dear Dr. Kumakech,

Thank you for submitting your manuscript to PLOS ONE. After careful consideration, we feel that it has merit but does not fully meet PLOS ONE’s publication criteria as it currently stands. Therefore, we invite you to submit a revised version of the manuscript that addresses the points raised during the review process.

Please submit your revised manuscript by Jan 01 2026 11:59PM. If you will need more time than this to complete your revisions, please reply to this message or contact the journal office at plosone@plos.org. When you’re ready to submit your revision, log on to https://www.editorialmanager.com/pone/ and select the ‘Submissions Needing Revision’ folder to locate your manuscript file.

Please include the following items when submitting your revised manuscript:

- A rebuttal letter that responds to each point raised by the academic editor and reviewer(s). You should upload this letter as a separate file labeled ‘Response to Reviewers’.
- A marked-up copy of your manuscript that highlights changes made to the original version. You should upload this as a separate file labeled ‘Revised Manuscript with Track Changes’.
- An unmarked version of your revised paper without tracked changes. You should upload this as a separate file labeled ‘Manuscript’.

If you would like to make changes to your financial disclosure, please include your updated statement in your cover letter. Guidelines for resubmitting your figure files are available below the reviewer comments at the end of this letter.

If applicable, we recommend that you deposit your laboratory protocols in protocols.io to enhance the reproducibility of your results. Protocols.io assigns your protocol its own identifier (DOI) so that it can be cited independently in the future. For instructions see: https://journals.plos.org/plosone/s/submission-guidelines#loc-laboratory-protocols. Additionally, PLOS ONE offers an option for publishing peer-reviewed Lab Protocol articles, which describe protocols hosted on protocols.io. Read more information on sharing protocols at https://plos.org/protocols?utm_medium=editorial-email&utm_source=authorletters&utm_campaign=protocols.

We look forward to receiving your revised manuscript.

Kind regards,

Hannah Mafo Degge, Ph.D

Academic Editor

PLOS ONE

Journal requirements:

When submitting your revision, we need you to address these additional requirements.

1. Please ensure that your manuscript meets PLOS ONE’s style requirements, including those for file naming. The PLOS ONE style templates can be found at

https://journals.plos.org/plosone/s/file?id=wjVg/PLOSOne_formatting_sample_main_body.pdf and

https://journals.plos.org/plosone/s/file?id=ba62/PLOSOne_formatting_sample_title_authors_affiliations.pdf

2. Your ethics statement should only appear in the Methods section of your manuscript. If your ethics statement is written in any section besides the Methods, please move it to the Methods section and delete it from any other section. Please ensure that your ethics statement is included in your manuscript, as the ethics statement entered into the online submission form will not be published alongside your manuscript.

3. If the reviewer comments include a recommendation to cite specific previously published works, please review and evaluate these publications to determine whether they are relevant and should be cited. There is no requirement to cite these works unless the editor has indicated otherwise.

Additional Editor Comments:

Introduction

- Address coherency issues in this section
- Lines 81-84 you wrote

*In countries like the United Kingdom (UK), a person living with HIV (PLHIV) is not permitted to*

*donate their eggs or sperm to another person or couple undergoing fertility treatment,*

*including their own friends and family (4).*

Does not match with statement before, as context is different – delete or use example from a similar context

- Lines 102- opening statement for paragraph, you wrote

*Despite the availability of ART and FP services and their reproductive health benefits for PLHIV,*

*little is known about the fertility aspirations of the YWLHIV in SSA settings such as Uganda, their*

*utilization of an integrated HIV care and FP services, and the factors influencing their*

*reproductive decisions is not appropriate as focus in on reproductive desires and factors*

Rephrase this paragraph to improve clarity and coherency. Consider splitting into two statements to address this

Discussion

Amend sentence line 488.. *The study revealed that an overwhelming majority (88.9%) of YWLHIV expressed a desire*

*The use of ‘overwhelming’ makes statement sound informal and subjective.*

Rather delete the word, and write. E.g *The study revealed that majority (88.9%) of YWLHIV expressed a desire…*

Work on critical analysis in your discussion section, for instance:

line 525 -527 you wrote

*The study also revealed that women with a positive history of a planned or intended pregnancy demonstrated 17% higher fertility desires (p=0.040). This finding concurs with a previous study conducted in southwestern Uganda (14). This suggest that access to conception or pregnancy planning may encourage childbearing intentions*

Paragraph is too short.  Consider the last statement in this paragraph and develop this further for critical analysis, for instance, what does this imply for YWLHIV, or why is this important to YWLHIV, what is the implication for integrated ART and FP services

Minor

Amend typo errors by deleting full stop in all your headings

*Line 533  and 538 you used the word  ‘don’t’*

Avoid contraction like ‘don’t in academic writing, change to ‘do not’ for a more formal writing

[Note: HTML markup is below. Please do not edit.]

Reviewers’ comments:

Reviewer’s Responses to Questions

**Comments to the Author**

1. Is the manuscript technically sound, and do the data support the conclusions?

The manuscript must describe a technically sound piece of scientific research with data that supports the conclusions. Experiments must have been conducted rigorously, with appropriate controls, replication, and sample sizes. The conclusions must be drawn appropriately based on the data presented.

Reviewer #3: Yes

Reviewer #4: Yes

**********

2. Has the statistical analysis been performed appropriately and rigorously?

Reviewer #3: Yes

Reviewer #4: No

**********

3. Have the authors made all data underlying the findings in their manuscript fully available?

The PLOS Data policy requires authors to make all data underlying the findings described in their manuscript fully available without restriction, with rare exception (please refer to the Data Availability Statement in the manuscript PDF file). The data should be provided as part of the manuscript or its supporting information, or deposited to a public repository. For example, in addition to summary statistics, the data points behind means, medians and variance measures should be available. If there are restrictions on publicly sharing data—e.g. participant privacy or use of data from a third party—those must be specified.

Reviewer #3: No

Reviewer #4: No

**********

4. Is the manuscript presented in an intelligible fashion and written in standard English?

PLOS ONE does not copyedit accepted manuscripts, so the language in submitted articles must be clear, correct, and unambiguous. Any typographical or grammatical errors should be corrected at revision, so please note any specific errors here.

Reviewer #3: No

Reviewer #4: No

**********

5. Review Comments to the Author

Please use the space provided to explain your answers to the questions above. You may also include additional comments for the author, including concerns about dual publication, research ethics, or publication ethics. (Please upload your review as an attachment if it exceeds 20,000 characters)

Reviewer #3: 1. General comments: very interesting study. It would seem there is a dearth of literature in this area.

2. Introduction Section: Too long and winding, comprehension is difficult.

3. Methodology Section:

a. Outcome Variable section of the article:

i. Authors have defined the term “fertility desires” in the Outcome Variable section of the Methodology. However, it will be better to being the early into the Introduction for better comprehension of the paper.

ii. Under the Outcome Variable section, the sentence “Accordingly, women whether they desire to conceive and produce more children despite their HIV positive status and reproductive history.” is not complete and should be re-phrased.

iii. This sentence needs to be rephrased and English improved for better comprehension “The women who expressed desire to have children where further asked about their desired number of children and also whether they male partner also desired to have children.”

b. Explanatory Variable section:

i. Authors should use “did not” instead of “didn’t” on line 255.

4. Results Section:

a. Table 2, authors should replace “address” with “residency” or perhaps “location”.

b. Authors are recommended to include the fertility desires and Integration of HIV and FP services into the Table 2 to improve comprehension.

i. In general, one is not sure which narrative is for which table. After reading I realized that if only significant variables were included in Table 3 while non-significant variables were described below. However there is an inconsistent arrangement in the narratives and tables, making it difficult to understand the Results section.

c. Authors should desist using acronyms such as “ vs.” in the write up

d. Thefollowing variables are not in Table 3.

i. “Women attending ART clinic facilities without FP services were less likely to desire children (85.6%) (p=0.044)”.

ii. “Drug users had a sharply lower desire for children (40.0%) (p=0.011).”

e. In general, authors should ensure that the narratives of tables actually reflect what is in the tables.

5. Discussion:

a. The authors should explain how does “The study finding concurs with previous conducted in Ethiopia where the utilization of an integrated HIV and FP services was found at 55.8% ..”

b. The authors should proffer possible reasons why their findings concur or do not align with literature with supporting references.

c. Authors should replace “don’t” with “do not”

6. Study Strength

a. The authors check the language

7. Conclusion

a. Authors mention semi-rural in the conclusion but assessed for YWLHIV in urban, semi-urban and rural areas in the results section. What is the reason for the discrepancy

Reviewer #4: The manuscript explores fertility desires and utilization of integrated HIV and family planning (FP) services among young women living with HIV (YWLHIV) in semi-rural Uganda. The topic is timely, contextually relevant, and contributes meaningfully to the growing body of literature on reproductive health integration within HIV programs in sub-Saharan Africa. While the study is conceptually sound and the sample size adequate, the manuscript requires major revisions to improve methodological clarity, statistical reporting accuracy, and depth of interpretation to enhance scientific rigour.

1. Study Design and Methodology:

1.a) The cross-sectional design is appropriate for the stated objectives. However, details on sampling procedures are limited. The selection criteria for ART clinics, proportional allocation of participants, and handling of non-respondents should be clearly described to assess representativeness.

1.b) Clarify how the questionnaire was validated, translated, and pre-tested. Since fertility desire is the central outcome, the exact question wording should appear in the Supplementary Material.

1.c) The section on ethics should be expanded to include institutional review board approval reference, consent procedures (particularly for minors), and data confidentiality measures.

2. Data Management and Statistical Analysis:

2.a) The use of modified Poisson regression is methodologically appropriate; however, reporting is inconsistent. Both crude and adjusted prevalence ratios (PRs) with 95% confidence intervals should be presented for all predictors in a single comprehensive table.

2.b) Clarify discrepancies between text and tables (e.g., Table 4 shows N = 103 whereas the overall sample is n = 423). Explain whether this refers to a subset or correct it if erroneous.

2.c) The discussion of multicollinearity diagnostics is inaccurate. Acceptable thresholds are typically tolerance ≥ 0.1 and VIF ≤ 5. Provide the actual VIF values per variable and correct the explanation accordingly.

2.d) Differentiate clearly between bivariate and multivariable findings. Avoid interpreting non-significant results as meaningful associations.

3. Results and Interpretation:

3.a) The high fertility desire (88.9%) is striking and should be contextualized. Discuss how cultural expectations, marital norms, and optimism about ART access might influence reproductive intentions among YWLHIV.

3.b) Interpret differences in FP utilization between those with and without fertility desires cautiously, acknowledging self-report and cross-sectional limitations.

3.c) Include a concise statement on model fit, handling of missing data, and sensitivity checks.

4. Discussion and Conclusions:

4.a) The discussion currently summarizes results but lacks analytical depth. Strengthen this section by comparing findings with similar studies from other East African countries (e.g., Kenya, Malawi, Tanzania) and discussing contextual determinants such as gender roles, stigma, and health system barriers.

4.b) Revise all causal language to reflect associations only (e.g., “factors associated with” rather than “predictors of”).

5. Presentation and Writing Quality:

5.a) The manuscript is generally clear but requires language editing for grammar, tense, and precision.

5.b) Use the term “integrated HIV and FP services” consistently throughout.

5.c) Ensure uniform presentation of p-values, percentages, and confidence intervals across text and tables.

5.d) Add a Data Availability Statement and deposit anonymized data and analysis code in a public repository (e.g., Zenodo, OSF, or Figshare), following PLOS ONE’s open data policy.

**********

6. PLOS authors have the option to publish the peer review history of their article (what does this mean?). If published, this will include your full peer review and any attached files.

If you choose “no”, your identity will remain anonymous but your review may still be made public.

**Do you want your identity to be public for this peer review?**  For information about this choice, including consent withdrawal, please see our Privacy Policy.

Reviewer #3: No

Reviewer #4: No

**********

[NOTE: If reviewer comments were submitted as an attachment file, they will be attached to this email and accessible via the submission site. Please log into your account, locate the manuscript record, and check for the action link "View Attachments". If this link does not appear, there are no attachment files.]

To ensure your figures meet our technical requirements, please review our figure guidelines: https://journals.plos.org/plosone/s/figures

You may also use PLOS’s free figure tool, NAAS, to help you prepare publication quality figures: https://journals.plos.org/plosone/s/figures#loc-tools-for-figure-preparation.

NAAS will assess whether your figures meet our technical requirements by comparing each figure against our figure specifications.

# Author comment: Author response to Decision Letter 1

**DOI:** 10.1371/journal.pone.0349072.r002

9 Jan 2026

We have responded to the specific reviewer and editor comments. We have attached it as Response to Reviewer 2 document.

Attachment Submitted filename: RESPONSES TO REVIEWERS.pdf

# Peer review: Decision Letter 1

**DOI:** 10.1371/journal.pone.0349072.r003

**Authors:** Hannah Mafo Degge

18 Feb 2026

PONE-D-25-45727R1

Fertility desires and utilization of an integrated HIV care and family planning services among young women living with HIV in semi-rural Uganda. A cross sectional study.

PLOS One

Dear Dr. Kumakech,

Thank you for submitting your manuscript to PLOS ONE. After careful consideration, we feel that it has merit but does not fully meet PLOS ONE’s publication criteria as it currently stands. Therefore, we invite you to submit a revised version of the manuscript that addresses the points raised during the review process.

Please submit your revised manuscript by Apr 04 2026 11:59PM. If you will need more time than this to complete your revisions, please reply to this message or contact the journal office at plosone@plos.org. When you’re ready to submit your revision, log on to https://www.editorialmanager.com/pone/ and select the ‘Submissions Needing Revision’ folder to locate your manuscript file.

Please include the following items when submitting your revised manuscript:

- A letter that responds to each point raised by the academic editor and reviewer(s). You should upload this letter as a separate file labeled ‘Response to Reviewers’.
- A marked-up copy of your manuscript that highlights changes made to the original version. You should upload this as a separate file labeled ‘Revised Manuscript with Track Changes’.
- An unmarked version of your revised paper without tracked changes. You should upload this as a separate file labeled ‘Manuscript’.

-->If you would like to make changes to your financial disclosure, please include your updated statement in your cover letter. Guidelines for resubmitting your figure files are available below the reviewer comments at the end of this letter.

If applicable, we recommend that you deposit your laboratory protocols in protocols.io to enhance the reproducibility of your results. Protocols.io assigns your protocol its own identifier (DOI) so that it can be cited independently in the future. For instructions see: https://journals.plos.org/plosone/s/submission-guidelines#loc-laboratory-protocols. Additionally, PLOS ONE offers an option for publishing peer-reviewed Lab Protocol articles, which describe protocols hosted on protocols.io. Read more information on sharing protocols at https://plos.org/protocols?utm_medium=editorial-email&utm_source=authorletters&utm_campaign=protocols.

We look forward to receiving your revised manuscript.

Kind regards,

Helen Howard

Staff Editor

PLOS One

Journal Requirements:

1. If the reviewer comments include a recommendation to cite specific previously published works, please review and evaluate these publications to determine whether they are relevant and should be cited. There is no requirement to cite these works unless the editor has indicated otherwise.

2. Please review your reference list to ensure that it is complete and correct. If you have cited papers that have been retracted, please include the rationale for doing so in the manuscript text, or remove these references and replace them with relevant current references. Any changes to the reference list should be mentioned in the rebuttal letter that accompanies your revised manuscript. If you need to cite a retracted article, indicate the article’s retracted status in the References list and also include a citation and full reference for the retraction notice.

Additional Editor Comments:

- Please note that PLOS ONE does not copyedit manuscripts, so the language in submitted articles must be clear, correct, and unambiguous. Please work to improve the quality of the writing throughout your manuscript. We recommend enlisting the help of a professional copyediting service.

[Note: HTML markup is below. Please do not edit.]

Reviewers’ comments:

Reviewer’s Responses to Questions

**Comments to the Author**

1. If the authors have adequately addressed your comments raised in a previous round of review and you feel that this manuscript is now acceptable for publication, you may indicate that here to bypass the “Comments to the Author” section, enter your conflict of interest statement in the “Confidential to Editor” section, and submit your "Accept" recommendation.

Reviewer #2: All comments have been addressed

Reviewer #4: All comments have been addressed

**********

2. Is the manuscript technically sound, and do the data support the conclusions?

The manuscript must describe a technically sound piece of scientific research with data that supports the conclusions. Experiments must have been conducted rigorously, with appropriate controls, replication, and sample sizes. The conclusions must be drawn appropriately based on the data presented.

Reviewer #2: Yes

Reviewer #4: Yes

**********

3. Has the statistical analysis been performed appropriately and rigorously?

Reviewer #2: (No Response)

Reviewer #4: Yes

**********

4. Have the authors made all data underlying the findings in their manuscript fully available?

The PLOS Data policy requires authors to make all data underlying the findings described in their manuscript fully available without restriction, with rare exception (please refer to the Data Availability Statement in the manuscript PDF file). The data should be provided as part of the manuscript or its supporting information, or deposited to a public repository. For example, in addition to summary statistics, the data points behind means, medians and variance measures should be available. If there are restrictions on publicly sharing data—e.g. participant privacy or use of data from a third party—those must be specified.

Reviewer #2: Yes

Reviewer #4: Yes

**********

5. Is the manuscript presented in an intelligible fashion and written in standard English?

PLOS ONE does not copyedit accepted manuscripts, so the language in submitted articles must be clear, correct, and unambiguous. Any typographical or grammatical errors should be corrected at revision, so please note any specific errors here.

Reviewer #2: Yes

Reviewer #4: Yes

**********

6. Review Comments to the Author

Please use the space provided to explain your answers to the questions above. You may also include additional comments for the author, including concerns about dual publication, research ethics, or publication ethics. (Please upload your review as an attachment if it exceeds 20,000 characters)

Reviewer #2: (No Response)

Reviewer #4: The authors have adequately addressed the concerns raised in the previous review. The study design and methodology are now clearly described, including sampling procedures, participant recruitment, proportional allocation, and management of non-response. The development of the questionnaire has been clarified, with details on validation, translation, and pre-testing, and the exact wording of the fertility desire outcome has been provided in the Supplementary Material. Ethical approval, consent procedures (including for minors), and data confidentiality measures are appropriately documented.

The statistical analysis is now clearly and consistently reported. The use of modified Poisson regression is appropriate, and both crude and adjusted prevalence ratios with 95% confidence intervals are presented in a single table. Earlier inconsistencies in sample size reporting have been resolved. The explanation of multicollinearity has been corrected using accepted thresholds, with relevant diagnostic values reported. The authors clearly distinguish between bivariate and multivariable results and avoid causal language, which is appropriate given the cross-sectional design.

The Results and Discussion have been strengthened by better contextualization of the high fertility desire among young women living with HIV, including discussion of sociocultural norms, marital expectations, and access to ART, and by comparison with findings from other East African settings. Policy and programmatic implications are clearly stated. The manuscript now meets PLOS ONE requirements for clarity, reporting, and data availability.

**********

7. PLOS authors have the option to publish the peer review history of their article (what does this mean?). If published, this will include your full peer review and any attached files.

If you choose “no”, your identity will remain anonymous but your review may still be made public.

**Do you want your identity to be public for this peer review?**  For information about this choice, including consent withdrawal, please see our Privacy Policy.

Reviewer #2: No

Reviewer #4: No

**********

[NOTE: If reviewer comments were submitted as an attachment file, they will be attached to this email and accessible via the submission site. Please log into your account, locate the manuscript record, and check for the action link "View Attachments". If this link does not appear, there are no attachment files.]

To ensure your figures meet our technical requirements, please review our figure guidelines: https://journals.plos.org/plosone/s/figures

You may also use PLOS’s free figure tool, NAAS, to help you prepare publication quality figures: https://journals.plos.org/plosone/s/figures#loc-tools-for-figure-preparation.

NAAS will assess whether your figures meet our technical requirements by comparing each figure against our figure specifications.

# Author comment: Author response to Decision Letter 2

**DOI:** 10.1371/journal.pone.0349072.r004

20 Mar 2026

RESPONSE TO THE REVIEWERS 3

COMMENTS RESPONSES

Editor’s Comments

1. If the reviewer comments include a recommendation to cite specific previously published works, please review and evaluate these publications to determine whether they are relevant and should be cited. There is no requirement to cite these works unless the editor has indicated otherwise. There were no reviewer’s recommendations to cite specific previously published works.

2. Please review your reference list to ensure that it is complete and correct. If you have cited papers that have been retracted, please include the rationale for doing so in the manuscript text, or remove these references and replace them with relevant current references. Any changes to the reference list should be mentioned in the rebuttal letter that accompanies your revised manuscript. If you need to cite a retracted article, indicate the article’s retracted status in the References list and also include a citation and full reference for the retraction notice. I conducted a systematic check by searching for each article title and its authors in the Retraction Watch Database, the most comprehensive and reliable source for this information. For the 46 specific titles you provided, I found no corresponding retraction notices or entries indicating they have been withdrawn.

I also checked the publisher’s website for updated editions or corrigenda for reports and data from organizations like references 1, 2 and 17 and found no updated reports as yet.

3. Additional Editor Comments:

- Please note that PLOS ONE does not copyedit manuscripts, so the language in submitted articles must be clear, correct and unambiguous. Please work to improve the quality of the writing throughout your manuscript. We recommend enlisting the help of a professional copyediting service. We enlisted the help of a professional copyediting service, and ensured the language in the submitted articles are clear, correct and unambiguous.

Reviewer’s Comments

Reviewer #4: The authors have adequately addressed the concerns raised in the previous review. The study design and methodology are now clearly described, including sampling procedures, participant recruitment, proportional allocation, and management of non-response. The development of the questionnaire has been clarified, with details on validation, translation, and pre-testing, and the exact wording of the fertility desire outcome has been provided in the Supplementary Material. Ethical approval, consent procedures (including for minors), and data confidentiality measures are appropriately documented.

The statistical analysis is now clearly and consistently reported. The use of modified Poisson regression is appropriate, and both crude and adjusted prevalence ratios with 95% confidence intervals are presented in a single table. Earlier inconsistencies in sample size reporting have been resolved. The explanation of multicollinearity has been corrected using accepted thresholds, with relevant diagnostic values reported. The authors clearly distinguish between bivariate and multivariable results and avoid causal language, which is appropriate given the cross-sectional design.

The Results and Discussion have been strengthened by better contextualization of the high fertility desire among young women living with HIV, including discussion of sociocultural norms, marital expectations, and access to ART, and by comparison with findings from other East African settings. Policy and programmatic implications are clearly stated. The manuscript now meets PLOS ONE requirements for clarity, reporting, and data availability. Thank you.

Attachment Submitted filename: RESPONSES TO REVIEWERS 3.pdf

# Editor report: Decision Letter 2

**DOI:** 10.1371/journal.pone.0349072.r005

**Authors:** Hannah Mafo Degge

30 Mar 2026

PONE-D-25-45727R2

Fertility desires and utilization of an integrated HIV care and family planning services among young women living with HIV in semi-rural Uganda. A cross sectional study.

PLOS One

Dear Dr. Kumakech,

Thank you for submitting your manuscript to PLOS ONE. After careful consideration, we feel that it has merit but does not fully meet PLOS ONE’s publication criteria as it currently stands. Therefore, we invite you to submit a revised version of the manuscript that addresses the points raised during the review process.

Please submit your revised manuscript by May 12 2026 11:59PM. If you will need more time than this to complete your revisions, please reply to this message or contact the journal office at plosone@plos.org. When you’re ready to submit your revision, log on to https://www.editorialmanager.com/pone/ and select the ‘Submissions Needing Revision’ folder to locate your manuscript file.

Please include the following items when submitting your revised manuscript:

- A letter that responds to each point raised by the academic editor and reviewer(s). You should upload this letter as a separate file labeled ‘Response to Reviewers’.
- A marked-up copy of your manuscript that highlights changes made to the original version. You should upload this as a separate file labeled ‘Revised Manuscript with Track Changes’.
- An unmarked version of your revised paper without tracked changes. You should upload this as a separate file labeled ‘Manuscript’.

If you would like to make changes to your financial disclosure, please include your updated statement in your cover letter. Guidelines for resubmitting your figure files are available below the reviewer comments at the end of this letter.

If applicable, we recommend that you deposit your laboratory protocols in protocols.io to enhance the reproducibility of your results. Protocols.io assigns your protocol its own identifier (DOI) so that it can be cited independently in the future. For instructions see: https://journals.plos.org/plosone/s/submission-guidelines#loc-laboratory-protocols. Additionally, PLOS ONE offers an option for publishing peer-reviewed Lab Protocol articles, which describe protocols hosted on protocols.io. Read more information on sharing protocols at https://plos.org/protocols?utm_medium=editorial-email&utm_source=authorletters&utm_campaign=protocols.

As the corresponding author, your ORCID iD is verified in the submission system and will appear in the published article. PLOS supports the use of ORCID, and we encourage all coauthors to register for an ORCID iD and use it as well. Please encourage your coauthors to verify their ORCID iD within the submission system before final acceptance, as unverified ORCID iDs will not appear in the published article. *Only*  the individual author can complete the verification step; PLOS staff *cannot*  verify ORCID iDs on behalf of authors.

We look forward to receiving your revised manuscript.

Kind regards,

Helen Howard

Staff Editor

PLOS One

Journal Requirements:

If the reviewer comments include a recommendation to cite specific previously published works, please review and evaluate these publications to determine whether they are relevant and should be cited. There is no requirement to cite these works unless the editor has indicated otherwise.

Please review your reference list to ensure that it is complete and correct. If you have cited papers that have been retracted, please include the rationale for doing so in the manuscript text, or remove these references and replace them with relevant current references. Any changes to the reference list should be mentioned in the rebuttal letter that accompanies your revised manuscript. If you need to cite a retracted article, indicate the article’s retracted status in the References list and also include a citation and full reference for the retraction notice.

Additional Editor Comments:

- We note that you have stated in your response document that you enlisted the help of a professional copyediting service, but it is unclear which copyediting service has been used, and language errors unfortunately remain throughout the manuscript. Currently the manuscript does not meet our criteria for publication, please see criterion #5 https://journals.plos.org/plosone/s/criteria-for-publication

Please work to improve the quality of the writing throughout your manuscript. We recommend enlisting the help of a professional copyediting service.

[Note: HTML markup is below. Please do not edit.]

[NOTE: If reviewer comments were submitted as an attachment file, they will be attached to this email and accessible via the submission site. Please log into your account, locate the manuscript record, and check for the action link "View Attachments". If this link does not appear, there are no attachment files.]

To ensure your figures meet our technical requirements, please review our figure guidelines: https://journals.plos.org/plosone/s/figures

You may also use PLOS’s free figure tool, NAAS, to help you prepare publication quality figures: https://journals.plos.org/plosone/s/figures#loc-tools-for-figure-preparation.

NAAS will assess whether your figures meet our technical requirements by comparing each figure against our figure specifications.

# Author comment: Author response to Decision Letter 3

**DOI:** 10.1371/journal.pone.0349072.r006

13 Apr 2026

COMMENTS RESPONSES

Editor’s Comments

1. If the reviewer comments include a recommendation to cite specific previously published works, please review and evaluate these publications to determine whether they are relevant and should be cited. There is no requirement to cite these works unless the editor has indicated otherwise. There were no reviewer’s recommendations to cite specific previously published works.

2. Please review your reference list to ensure that it is complete and correct. If you have cited papers that have been retracted, please include the rationale for doing so in the manuscript text, or remove these references and replace them with relevant current references. Any changes to the reference list should be mentioned in the rebuttal letter that accompanies your revised manuscript. If you need to cite a retracted article, indicate the article’s retracted status in the References list and also include a citation and full reference for the retraction notice. I conducted a systematic check by searching for each article title and its authors in the Retraction Watch Database, the most comprehensive and reliable source for this information. For the 46 specific titles you provided, I found no corresponding retraction notices or entries indicating they have been withdrawn.

I also checked the publisher’s website for updated editions or corrigenda for reports and data from organizations like references 1, 2 and 17 and found no updated reports as yet.

3. Additional Editor Comments:

- Please note that PLOS ONE does not copyedit manuscripts, so the language in submitted articles must be clear, correct and unambiguous. Please work to improve the quality of the writing throughout your manuscript. We recommend enlisting the help of a professional copyediting service. We enlisted the help of a professional copyediting service, and ensured the language in the submitted articles are clear, correct and unambiguous.

Attachment Submitted filename: RESPONSES TO REVIEWERS 4.pdf

# Peer review: Decision Letter 3

**DOI:** 10.1371/journal.pone.0349072.r007

**Authors:** Helen Howard

17 May 2026

PONE-D-25-45727R3

Fertility desires and utilization of an integrated HIV care and family planning services among young women living with HIV in semi-rural Uganda. A cross sectional study.

PLOS One

Dear Dr. Kumakech,

Thank you for submitting your manuscript to PLOS ONE. After careful consideration, we feel that it has merit but does not fully meet PLOS ONE’s publication criteria as it currently stands. Therefore, we invite you to submit a revised version of the manuscript that addresses the points raised during the review process.

Please submit your revised manuscript by Jun 29 2026 11:59PM. If you will need more time than this to complete your revisions, please reply to this message or contact the journal office at plosone@plos.org. When you’re ready to submit your revision, log on to https://www.editorialmanager.com/pone/ and select the ‘Submissions Needing Revision’ folder to locate your manuscript file.

Please include the following items when submitting your revised manuscript:

- A letter that responds to each point raised by the academic editor and reviewer(s). You should upload this letter as a separate file labeled ‘Response to Reviewers’.
- A marked-up copy of your manuscript that highlights changes made to the original version. You should upload this as a separate file labeled ‘Revised Manuscript with Track Changes’.
- An unmarked version of your revised paper without tracked changes. You should upload this as a separate file labeled ‘Manuscript’.

If you would like to make changes to your financial disclosure, please include your updated statement in your cover letter. Guidelines for resubmitting your figure files are available below the reviewer comments at the end of this letter.

If applicable, we recommend that you deposit your laboratory protocols in protocols.io to enhance the reproducibility of your results. Protocols.io assigns your protocol its own identifier (DOI) so that it can be cited independently in the future. For instructions see: https://journals.plos.org/plosone/s/submission-guidelines#loc-laboratory-protocols. Additionally, PLOS ONE offers an option for publishing peer-reviewed Lab Protocol articles, which describe protocols hosted on protocols.io. Read more information on sharing protocols at https://plos.org/protocols?utm_medium=editorial-email&utm_source=authorletters&utm_campaign=protocols.

As the corresponding author, your ORCID iD is verified in the submission system and will appear in the published article. PLOS supports the use of ORCID, and we encourage all coauthors to register for an ORCID iD and use it as well. Please encourage your coauthors to verify their ORCID iD within the submission system before final acceptance, as unverified ORCID iDs will not appear in the published article. *Only*  the individual author can complete the verification step; PLOS staff *cannot*  verify ORCID iDs on behalf of authors.

We look forward to receiving your revised manuscript.

Kind regards,

Helen Howard

Staff Editor

PLOS One

Journal Requirements:

1. If the reviewer comments include a recommendation to cite specific previously published works, please review and evaluate these publications to determine whether they are relevant and should be cited. There is no requirement to cite these works unless the editor has indicated otherwise.

2. Please review your reference list to ensure that it is complete and correct. If you have cited papers that have been retracted, please include the rationale for doing so in the manuscript text, or remove these references and replace them with relevant current references. Any changes to the reference list should be mentioned in the rebuttal letter that accompanies your revised manuscript. If you need to cite a retracted article, indicate the article’s retracted status in the References list and also include a citation and full reference for the retraction notice.

Additional Editor Comments:

Unfortunately we remain concerned by language errors throughout the manuscript. Whilst copyediting was carried out previously, it seems that errors/inconsistencies may have been introduced during the revision process. Some examples are:

1) Line 441 states that the majority of participants were married, but the results in Table 2 indicate that most were single (52% single vs 48% married).

2) We note that on line 457 (and others), the frequency and 95% CI is not presented in a standard format. The correct format is given on line 469 [i.e. 51 (12.1%, 95% CI 9.0 – 15.2%)]. Please ensure that this is how the frequency and 95% CI is stated throughout the manuscript, rather than [376 (88.9%) with a 95% CI of 85.6% –91.6%].

3) There are other copyediting errors/inconsistencies throughout the manuscript. Please ensure a thorough revision, or enlist the help of a professional copyeditor, to correct these and improve the quality of the writing.

[Note: HTML markup is below. Please do not edit.]

Reviewers’ comments:

Reviewer’s Responses to Questions

**Comments to the Author**

1. If the authors have adequately addressed your comments raised in a previous round of review and you feel that this manuscript is now acceptable for publication, you may indicate that here to bypass the “Comments to the Author” section, enter your conflict of interest statement in the “Confidential to Editor” section, and submit your "Accept" recommendation.

Reviewer #5: All comments have been addressed

**********

2. Is the manuscript technically sound, and do the data support the conclusions?

The manuscript must describe a technically sound piece of scientific research with data that supports the conclusions. Experiments must have been conducted rigorously, with appropriate controls, replication, and sample sizes. The conclusions must be drawn appropriately based on the data presented.

Reviewer #5: Yes

**********

3. Has the statistical analysis been performed appropriately and rigorously?

Reviewer #5: Yes

**********

4. Have the authors made all data underlying the findings in their manuscript fully available?

The PLOS Data policy requires authors to make all data underlying the findings described in their manuscript fully available without restriction, with rare exception (please refer to the Data Availability Statement in the manuscript PDF file). The data should be provided as part of the manuscript or its supporting information, or deposited to a public repository. For example, in addition to summary statistics, the data points behind means, medians and variance measures should be available. If there are restrictions on publicly sharing data—e.g. participant privacy or use of data from a third party—those must be specified.

Reviewer #5: Yes

**********

5. Is the manuscript presented in an intelligible fashion and written in standard English?

PLOS ONE does not copyedit accepted manuscripts, so the language in submitted articles must be clear, correct, and unambiguous. Any typographical or grammatical errors should be corrected at revision, so please note any specific errors here.

Reviewer #5: Yes

**********

6. Review Comments to the Author

Please use the space provided to explain your answers to the questions above. You may also include additional comments for the author, including concerns about dual publication, research ethics, or publication ethics. (Please upload your review as an attachment if it exceeds 20,000 characters)

Reviewer #5: Without formal hypothesis statements and a power considerations the investigators used the Kish Leslie formula for sample size determination. The assumptions included a 50% prevalence, 1.96 standard deviation corresponding with 95% confidence level from the standard normal distribution curve and a 0.05 margin of error. This gave the minimum required sample size of YWLHIV. This initial sample size was further adjusted for a 10% non-response rate resulting into a final target sample size of 423 YWLHIV.

The structured questionnaire and explanatory and confounding effects were reasonably considered, The data management and analysis were targeted for mostly a descriptive type of presentation with some inferential considerations..

Descriptive statistics, parametric and non parametric, were used to summarize the fertility desire rate and the participant’s characteristics were the frequency counts, percentages, mean, standard deviations (SD), median and the interquartile range (IQR). Associations between independent and outcome variables were assessed using the Chi-square or the Fisher’s Exact Tests. Factors showing significant associations at the bivariate analysis were first assessed for multi-collinearity before entry into a multivariable regression analysis. The analysis was simple overall and the progression from bivariate to multiple regression with the appropriate strategies including the appropriate models applied were easily followed. The tables were well formatted and easily presented. The conclusions followed from the analyses performed.

The progression of the 3 revisions appeared reasonable as per the tolerances of the statistical boundary cutoffs as per the previous reviewers. The investigators responded appropriately. The added note from the third reviewer that the statistical analysis is now clearly and consistently reported is certainly correct. The added use of modified Poisson regression is appropriate, and both crude and adjusted prevalence ratios with 95% confidence intervals are presented in an appropriate format as noted.

**********

7. PLOS authors have the option to publish the peer review history of their article (what does this mean?). If published, this will include your full peer review and any attached files.

If you choose “no”, your identity will remain anonymous but your review may still be made public.

**Do you want your identity to be public for this peer review?**  For information about this choice, including consent withdrawal, please see our Privacy Policy.

Reviewer #5: No

**********

[NOTE: If reviewer comments were submitted as an attachment file, they will be attached to this email and accessible via the submission site. Please log into your account, locate the manuscript record, and check for the action link "View Attachments". If this link does not appear, there are no attachment files.]

To ensure your figures meet our technical requirements, please review our figure guidelines: https://journals.plos.org/plosone/s/figures

You may also use PLOS’s free figure tool, NAAS, to help you prepare publication quality figures: https://journals.plos.org/plosone/s/figures#loc-tools-for-figure-preparation.

NAAS will assess whether your figures meet our technical requirements by comparing each figure against our figure specifications.

# Author comment: Author response to Decision Letter 4

**DOI:** 10.1371/journal.pone.0349072.r008

22 Jun 2026

RESPONSE TO REVIEWERS

COMMENTS RESPONSE

If the reviewer comments include a recommendation to cite specific previously published works, please review and evaluate these publications to determine whether they are relevant and should be cited. There is no requirement to cite these works unless the editor has indicated otherwise. Not applicable.

Please review your reference list to ensure that it is complete and correct. If you have cited papers that have been retracted, please include the rationale for doing so in the manuscript text, or remove these references and replace them with relevant current references. Any changes to the reference list should be mentioned in the rebuttal letter that accompanies your revised manuscript. If you need to cite a retracted article, indicate the article’s retracted status in the References list and also include a citation and full reference for the retraction notice. The reference list is reviewed, complete and correct

1) Line 441 states that the majority of participants were married, but the results in Table 2 indicate that most were single (52% single vs 48% married). In line 441, we have corrected that the majority of the participants were single.

2) We note that on line 457 (and others), the frequency and 95% CI is not presented in a standard format. The correct format is given on line 469 [i.e. 51 (12.1%, 95% CI 9.0 – 15.2%)]. Please ensure that this is how the frequency and 95% CI is stated throughout the manuscript, rather than [376 (88.9%) with a 95% CI of 85.6% –91.6%]. In lines 457 – 465, we have used the correct format to presented the frequency count, percent and 95% CI.

3) There are other copyediting errors/inconsistencies throughout the manuscript. Please ensure a thorough revision, or enlist the help of a professional copyeditor, to correct these and improve the quality of the writing. We have enlisted the help of a professional copyeditor to correct all the copyediting errors/ inconsistencies throughout the manuscript.

Reviewer #5: Without formal hypothesis statements and a power consideration the investigators used the Kish Leslie formula for sample size determination. The assumptions included a 50% prevalence, 1.96 standard deviation corresponding with 95% confidence level from the standard normal distribution curve and a 0.05 margin of error. This gave the minimum required sample size of YWLHIV. This initial sample size was further adjusted for a 10% non-response rate resulting into a final target sample size of 423 YWLHIV. We thank the reviewer for this important methodological observation. We acknowledge that our initial sample size calculation, based on the Kish Leslie formula, was indeed designed to estimate a prevalence (descriptive objective) with a desired precision (5%), rather than to test a specific a priori hypothesis for inferential comparisons.

We recognize that, ideally, sample size should be determined by power calculations tied to the formal hypothesis statements for the primary analytical comparisons. However, given the exploratory nature of this study and the lack of prior published data on key associations within this specific population of YWLHIV in northern Uganda setting, we prioritized obtaining a sufficiently large and precise descriptive estimate. The final achieved sample of 423 YWLHIV was a pragmatic balance between statistical precision and logistical feasibility.

To address concerns regarding analytical power, we conducted a post-hoc sensitivity analysis to evaluate the minimum detectable effect sizes for our secondary/ comparative analyses at 80% power and α=0.05, with our achieved sample of 423 participants:

• For estimating a single proportion, our 95% confidence intervals around any observed prevalence have a maximum width of approximately ±4.8%, confirming that our precision objective was met.

• For comparing two independent subgroups of roughly equal size, our sample had 80% power to detect a difference of approximately 12–13 percentage points between the groups.

• For a logistic regression model (if applicable), our sample size of 423 comfortably satisfies the standard ‘events-per-variable’ rule of thumb (at least 10–15 events per predictor), allowing us to fit a stable multivariable model."

We have now clearly stated this as a sample size determination in the revised manuscript (see lines 217-233).

Therefore, while we agree that formal a priori power calculations for each hypothesis would have strengthened the study design, we are confident that the final sample size of 423 provides adequate precision for our primary descriptive aim and sufficient sensitivity to detect clinically meaningful differences in our exploratory analyses.

We have now clearly stated this as a limitation in the revised manuscript (see lines 710-714).

The structured questionnaire and explanatory and confounding effects were reasonably considered, The data management and analysis were targeted for mostly a descriptive type of presentation with some inferential considerations..

Descriptive statistics, parametric and non- parametric, were used to summarize the fertility desire rate and the participant’s characteristics were the frequency counts, percentages, mean, standard deviations (SD), median and the interquartile range (IQR). Associations between independent and outcome variables were assessed using the Chi-square or the Fisher’s Exact Tests. Factors showing significant associations at the bivariate analysis were first assessed for multi-collinearity before entry into a multivariable regression analysis. The analysis was simple overall and the progression from bivariate to multiple regression with the appropriate strategies including the appropriate models applied were easily followed. The tables were well formatted and easily presented. The conclusions followed from the analyses performed.

The progression of the 3 revisions appeared reasonable as per the tolerances of the statistical boundary cutoffs as per the previous reviewers. The investigators responded appropriately. The added note from the third reviewer that the statistical analysis is now clearly and consistently reported is certainly correct. The added use of modified Poisson regression is appropriate, and both crude and adjusted prevalence ratios with 95% confidence intervals are presented in an appropriate format as noted. We are deeply grateful to the reviewer for the thorough and positive evaluation of our statistical methods and analysis.

We greatly appreciate the time and expertise invested in reviewing the progression of our revisions and the final analytical approach.

We are particularly encouraged by the reviewer’s confirmation that:

• The structured questionnaire and consideration of explanatory and confounding effects were reasonable;

• The descriptive and inferential analyses, including the use of parametric and non-parametric methods, were appropriate;

• The progression from bivariate analysis to multivariable regression, with appropriate checks for multi-collinearity and the use of modified Poisson regression, was clearly presented and methodologically sound;

• The presentation of both crude and adjusted prevalence ratios with 95% confidence intervals is appropriate; and

• The conclusions are consistent with the analyses performed.

We are pleased that the statistical reporting now meets the standards expected by the reviewer and the journal. We have carefully reviewed the manuscript once more to ensure that all statistical terminology is consistent throughout, and we have made minor editorial refinements to enhance clarity where possible (these are highlighted in the revised version).

We thank the reviewer again for their constructive engagement throughout this process, which has significantly strengthened the quality of our manuscript.

Attachment Submitted filename: RESPONSE TO REVIEWERS 5.pdf

# Editor report: Decision Letter 4

**DOI:** 10.1371/journal.pone.0349072.r009

**Authors:** Helen Howard

8 Jul 2026

Fertility desires and utilization of an integrated HIV care and family planning services among young women living with HIV in semi-rural Uganda. A cross sectional study.

PONE-D-25-45727R4

Dear Dr. Kumakech,

We’re pleased to inform you that your manuscript has been judged scientifically suitable for publication and will be formally accepted for publication once it meets all outstanding technical requirements.

Within one week, you’ll receive an e-mail detailing the required amendments. When these have been addressed, you’ll receive a formal acceptance letter and your manuscript will be scheduled for publication.

An invoice will be generated when your article is formally accepted. Please note, if your institution has a publishing partnership with PLOS and your article meets the relevant criteria, all or part of your publication costs will be covered. Please make sure your user information is up-to-date by logging into Editorial Manager at Editorial Manager® and clicking the ‘Update My Information’ link at the top of the page. For questions related to billing, please contact billing support.

If your institution or institutions have a press office, please notify them about your upcoming paper to help maximize its impact. If they’ll be preparing press materials, please inform our press team as soon as possible -- no later than 48 hours after receiving the formal acceptance. Your manuscript will remain under strict press embargo until 2 pm Eastern Time on the date of publication. For more information, please contact onepress@plos.org.

Kind regards,

Helen Howard

Staff Editor

PLOS One

Additional Editor Comments (optional):

Reviewers’ comments:

# Editor report: Acceptance letter

**DOI:** 10.1371/journal.pone.0349072.r010

**Authors:** Helen Howard

PONE-D-25-45727R4

PLOS One

Dear Dr. Kumakech,

I’m pleased to inform you that your manuscript has been deemed suitable for publication in PLOS One. Congratulations! Your manuscript is now being handed over to our production team.

At this stage, our production department will prepare your paper for publication. This includes ensuring the following:

* All references, tables, and figures are properly cited

* All relevant supporting information is included in the manuscript submission,

* There are no issues that prevent the paper from being properly typeset

You will receive further instructions from the production team, including instructions on how to review your proof when it is ready. Please keep in mind that we are working through a large volume of accepted articles, so please give us a few days to review your paper and let you know the next and final steps.

Lastly, if your institution or institutions have a press office, please let them know about your upcoming paper now to help maximize its impact. If they’ll be preparing press materials, please inform our press team within the next 48 hours. Your manuscript will remain under strict press embargo until 2 pm Eastern Time on the date of publication. For more information, please contact onepress@plos.org.

You will receive an invoice from PLOS for your publication fee after your manuscript has reached the completed accept phase. If you receive an email requesting payment before acceptance or for any other service, this may be a phishing scheme. Learn how to identify phishing emails and protect your accounts at https://explore.plos.org/phishing.

If we can help with anything else, please email us at customercare@plos.org.

Thank you for submitting your work to PLOS ONE and supporting open access.

Kind regards,

PLOS ONE Editorial Office Staff

on behalf of

Dr Helen Howard

Staff Editor

PLOS One
